# Targeting Endogenous Lipophagy: A Novel Strategy to Enhance MSC Osteogenesis and Mineralization for Senile Osteoporosis Therapy

**DOI:** 10.1002/advs.75348

**Published:** 2026-04-27

**Authors:** Chaoqiang Chen, Zhidong Liu, Yanhang Sun, Xiaojun Xu, Zhexiao Lan, Junhao Zhao, Peitao Xu, Guiwen Ye, Jinteng Li

**Affiliations:** ^1^ Department of Orthopedics The Eighth Affiliated Hospital ,Sun Yat‐Sen University Shenzhen China

**Keywords:** lipophagy, mesenchymal stem cells, mitochondrial bioenergetics, senile osteoporosis, SPARTIN

## Abstract

Senile osteoporosis (SOP) is characterized by impaired osteogenesis of bone‐marrow mesenchymal stem cells (MSCs). The underlying metabolic basis remains unclear. This study aimed to identify energy‐regulating pathways sustaining MSC osteogenesis during aging. Progressive activation of lipophagy was observed during MSC osteogenic differentiation, coupling lipid‐droplet degradation with mitochondrial β‐oxidation and ATP generation. Loss of the lipophagy receptor SPARTIN disrupted this process, leading to lipid accumulation, reduced CPT1A/CPT2 expression, suppressed oxidative phosphorylation, and impaired osteogenesis in vitro and in vivo. Conditional deletion of Spart in MSCs reproduced an osteoporosis‐like phenotype in young mice. Reactivation of lipophagy using bone‐tropic AAV9‐LAP restored mitochondrial metabolism and bone mass in both Spart‐CKO and SOP mice. Pharmacological activation with digoxin produced similar effects but induced cardiotoxicity. A senescent‐neutrophil‐membrane‐coated nanoplatform (SNM@NP‐DIG) enabled bone‐targeted digoxin delivery, rescuing bone mass while minimizing cardiac injury. Overall, SPARTIN‐mediated lipophagy is a critical metabolic regulator of MSC osteogenesis and represents a promising therapeutic target for senile osteoporosis.

## Graphical abstract

Schematic representation of the role of lipophagy in bone mesenchymal stem cells(MSCs). In healthy MSCs, functional lipophagy efficiently degrades lipid droplets to support oxidative phosphorylation and cellular energy production, thereby facilitating osteogenic differentiation and matrix mineralization. In contrast, aged mice exhibit impaired lipophagy, which results in reduced oxidative phosphorylation and subsequently weakened osteogenesis and mineralization capacity of MSCs, ultimately contributing to an osteoporotic phenotype. Importantly, restoring lipophagy in aged MSCs using a bone‐tropic AAV9 vector carrying the LAP construct (btAAV9‐LAP), digoxin (DIG), or senescent neutrophil membrane‐coated nanoparticles loaded with digoxin (SNM@NP‐DIG) effectively alleviates osteoporosis.

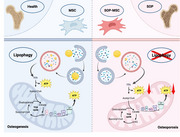

## 1 Introduction

The pathogenesis of senile osteoporosis (SOP) is characterized by impaired bone formation with relatively active bone resorption [1, 2, 3]. Thus, restoring the diminished osteogenic capacity of senescent mesenchymal stromal cells(MSCs) is crucial for SOP treatment [4, 5, 6]. Osteogenesis of MSCs and the synthesis and secretion of mineralized vesicles demand substantial cellular energy [7, 8, 9]. Consequently, metabolic reprogramming occurs, transitioning MSCs from glycolysis to oxidative phosphorylation, a pathway that provides substantial cellular energy [10]. However, previous studies have demonstrated dysfunction in the oxidative phosphorylation of SOP‐MSCs, significantly impairing their osteogenic capacity [11]. Nevertheless, the specific mechanisms underlying oxidative phosphorylation dysfunction in SOP‐MSCs remain unclear and require further elucidation.

Fatty acid oxidation provides essential substrates for mitochondrial oxidative phosphorylation, supplying critical sources of mitochondrial ATP and NADH. Lipid droplets are critical reservoirs of fatty acids within cells. Initial studies have shown increased synthesis of endogenous lipid droplets during osteoblast differentiation, which can subsequently be catabolized into fatty acids via canonical lipolysis, providing ATP to osteoblasts through fatty acid oxidation coupled to oxidative phosphorylation. It has been established that interference with endogenous lipolysis significantly inhibits ATP production, consequently impairing osteogenesis and mineralization [12]. In addition to classical lipolytic enzyme‐mediated lipolysis, recent studies have identified selective autophagy‐lysosomal‐mediated degradation of lipid droplets, termed lipophagy [13]. Early studies have demonstrated autophagy activation during BMSC osteogenesis and indicated that autophagy suppression disrupts osteogenesis and mineralization. Transmission electron microscopy (TEM) analyses have revealed increased autophagosome formation during osteogenic differentiation in osteoblastic cells [14]. Additionally, osteoblast‐specific ATG5 knockout mice exhibit an osteoporosis‐like phenotype [15]. Interestingly, enhanced mitophagy has been shown to rescue oxidative stress‐induced MSC senescence [16]. Although classical lipolysis and autophagy are known to regulate MSC osteogenesis and mineralization, whether selective lipophagy participates in these processes remains unclear and warrants further investigation.

While dysfunctions in lipolysis and autophagy during aging are well characterized, emerging evidence now directly links impaired lipophagy to age‐related pathologies. Enhanced lipophagy has been demonstrated to ameliorate non‐alcoholic fatty liver disease (NAFLD) and hepatocyte senescence [17]. In age‐related cirrhosis, suppression of lipophagy mediated by SCAD exacerbates both hepatic injury and cellular senescence [18]. However, whether SOP‐MSCs exhibit similar lipophagy dysfunction, potentially disrupting fatty acid oxidation‐oxidative phosphorylation coupling and impairing osteogenic capacity, remains unclear.

This study revealed for the first time that lipophagy plays an important role in MSCs osteogenic differentiation. These results demonstrated that lipophagy was activated during osteogenic differentiation, and its inhibition significantly suppressed β‐oxidation and oxidative phosphorylation in MSCs, thereby impairing osteogenesis and mineralization. Genetic ablation of the lipophagy receptor SPARTIN in MSCs induced an osteoporosis‐like phenotype in middle‐aged mice, thereby implicating the impaired lipophagy‐fatty acid oxidation‐mitochondrial oxidative phosphorylation (FAO‐OXPHOS) axis in the pathogenesis of senile osteoporosis. To counteract lipophagy impairment in SOP‐MSCs, a targeted therapeutic strategy employing bone marrow‐specific AAV9‐mediated delivery of a lipophagy‐enhancing molecule (LIR) and digoxin‐loaded nanoparticles was developed for selective activation of lipophagy in SOP‐MSCs. These interventions significantly restored FAO‐OXPHOS‐mediated ATP production and consequently rescued the osteogenic capacity of SOP‐MSCs, thus providing a promising therapeutic orientation for senile osteoporosis.

## 2 Results

### 2.1 Enhanced Lipophagic Activity in MSCs During the Late Stage of Osteogenic Differentiation

**FIGURE 1:**
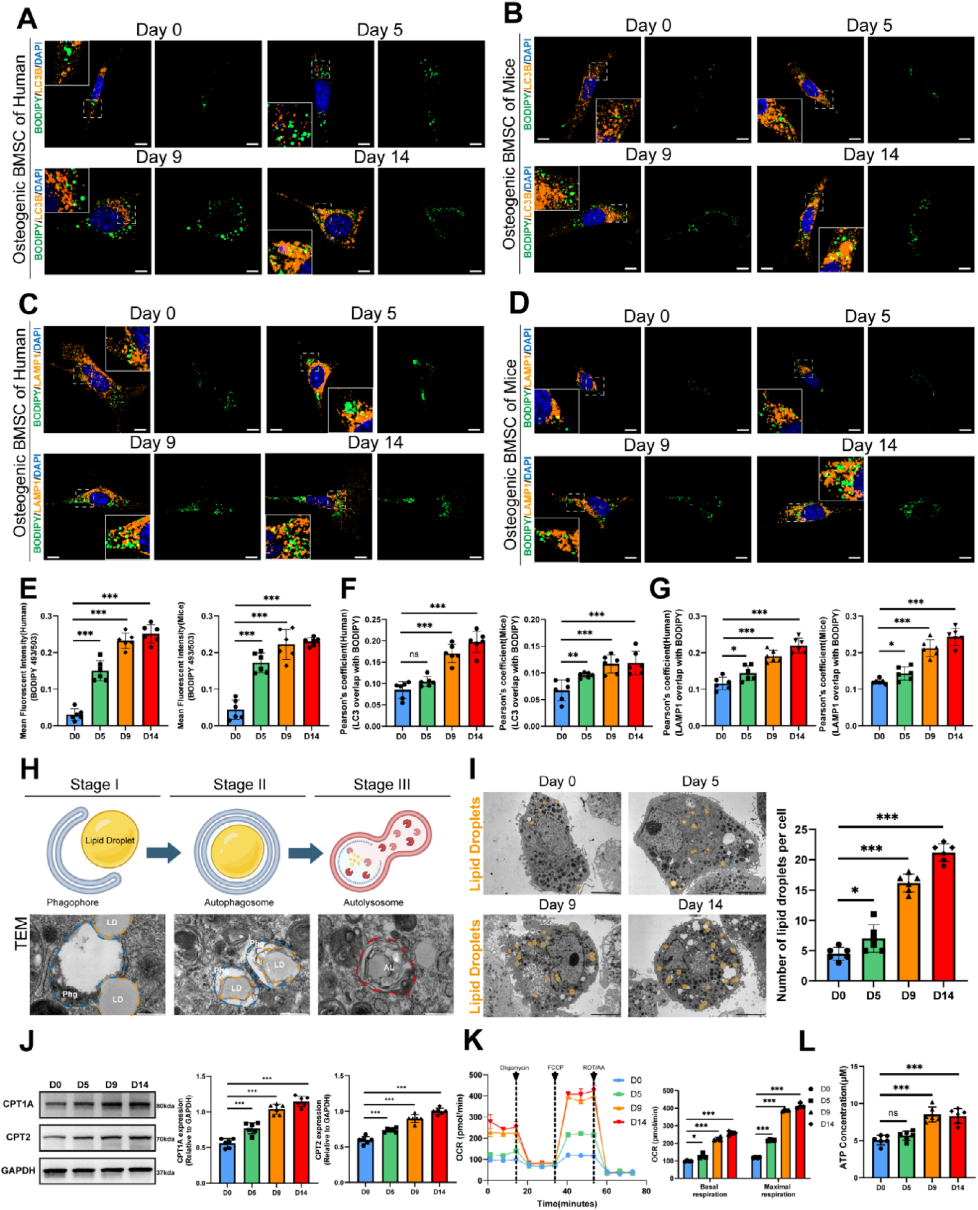
Enhanced lipophagic activity in MSCs during the late stage of osteogenic differentiation (A, B) Representative immunofluorescence micrographs of LC3 (orange) and BODIPY 493/503 (green) double‐staining to label autophagosomes and LDs in four different time stages of osteogenic differentiation of human(A) and mice(B) MSCs, respectively. (scale bar = 10 µm) (C, D) Representative immunofluorescence micrographs of LAMP1 (orange) and BODIPY 493/503 (green) double‐staining to label autolysosomes and LDs in four different time stages of osteogenic differentiation of human(C) and mice(D) MSCs, respectively. (scale bar = 10 µm) (E) Mean fluorescence intensity of BODIPY 493/503 (green) in four different time stages of osteogenic differentiation of human(left) and mice(right) MSCs, respectively. (*n =* 6). (F, G) Pearson's coefficient of four different time stages of osteogenic differentiation of human(left) and mice(right) MSCs, respectively, indicating the colocalization of autophagosomes(F) and autolysosomes(G) linking to LDs. (*n =* 6). (H) Schematic illustration (upper panel) and representative TEM images (lower panel) depict the sequential stages of lipophagy: (I) lipid droplets (yellow) approaching autophagosomes (blue), (II) engulfment of lipid droplets by autophagosomes, and (III) fusion of lipid droplet–containing autophagosomes with lysosomes to form lipolysosomes (red). scale bar = 2 µm (I) Representative TEM images showing intracellular lipid droplets (yellow) in MSCs at various stages of osteogenic differentiation, with quantification of lipid droplet abundance(right). scale bar = 500 nm (*n =* 6) (J) Representative immunoblots (left) and quantitative analysis (right) showing protein levels of the FAO key enzymes CPT1A and CPT2 in MSCs at different stages of osteogenic differentiation. (*n =* 6) (K) Curve and quantitative analysis of the OCRs for MSCs at different stages of osteogenic differentiation. (*n =* 6) (L) Quantification of intracellular ATP levels in MSCs at various stages of osteogenic differentiation. (*n =* 6) Differences among multiple groups were analyzed by one‐way ANOVA, followed by Bonferroni post‐hoc test for multiple comparisons, and the data are expressed as mean ± SD, *ns*: no significance; ^*^ p < 0.05, ^**^ p < 0.01, ^***^ p < 0.001.

To investigate the role of lipophagy during osteogenic differentiation of MSCs, MSCs were isolated from the bone marrow of human and mouse donors and induced osteogenesis in vitro. Cells were collected at multiple time points (Day 0, 5, 9, and 14) to monitor dynamic changes in lipophagic activity. During lipophagy, autophagosomes approach and interact with lipid droplets (LDs); therefore, colocalization of the autophagosomal marker LC3B with LDs was examined by immunofluorescence staining (Figure 1A,B). As the process progresses, LDs are engulfed by autolysosomes, resulting in colocalization of the lysosomal membrane protein LAMP1 with LDs (Figure 1C,D). Both colocalization patterns were quantified as indicators of lipophagic activity. Based on these analyses, intracellular LDs markedly increased with prolonged osteogenic differentiation in both human and mouse MSCs, particularly on days 9 and 14, when distinct LDs structures became apparent (Figure 1E). Concurrently, LC3B‐LDs and LAMP1‐LDs colocalization significantly increased, indicating enhanced lipophagic activity (Figure 1F,G). These findings suggest that lipid metabolism and lipophagy are strongly activated during the late stages of MSCs osteogenic differentiation. To further visualize lipophagy, TEM analysis was performed, which revealed sequential stages of the process: (I) formation of a double‐membrane autophagosome approaching and engulfing LDs; (II) completion of the autophagosome; and (III) fusion with lysosomes to generate autolysosomes (Figure 1H). TEM also confirmed gradual LDs accumulation during osteogenesis, although lipophagic structures were not captured in all sections due to the limitations of ultrathin slicing (Figure 1I). Consistently, intracellular triglyceride levels progressively increased during differentiation (Figure S1A).

Although these observations demonstrated that lipophagy increases during MSCs osteogenic differentiation, its impact on osteogenesis remained unclear. Lipophagy, an acidic form of lipolysis, degrades intracellular triglycerides within lysosomes, releasing free fatty acids (FFA). These FFA serve as high‐energy substrates that undergo mitochondrial FAO and OXPHOS to produce ATP, which is essential for MSCs at late differentiation stages. To test this mechanism, the intracellular FFA concentration was tested. Intracellular FFA levels progressively increased during differentiation (Figure S1B). Western blotting was subsequently performed to quantify the protein expression of CPT1A and CPT2—the rate‐limiting enzymes in FAO—both of which progressively increased during osteogenic differentiation (Figure 1J; Figure S1C,D), suggesting enhanced mitochondrial import of fatty acyl‐CoA derived from FFA. The mitochondrial OXPHOS was assessed by measuring the oxygen consumption rate (OCR). Consistent with increased FAO level, basal and maximal respiration both rose as differentiation progressed, and ATP production via OXPHOS was significantly elevated at later stages (Figure 1K; Figure S1E). Total intracellular ATP content likewise increased during osteogenic differentiation (Figure 1L).

Collectively, these results demonstrate that lipophagic activity in MSCs is progressively enhanced during osteogenic differentiation, especially at late stages, accompanied by increased FAO and OXPHOS, ultimately leading to elevated ATP generation.

### 2.2 Inhibition of Lipophagy Compromises MSC Osteogenic Differentiation In Vitro

**FIGURE 2:**
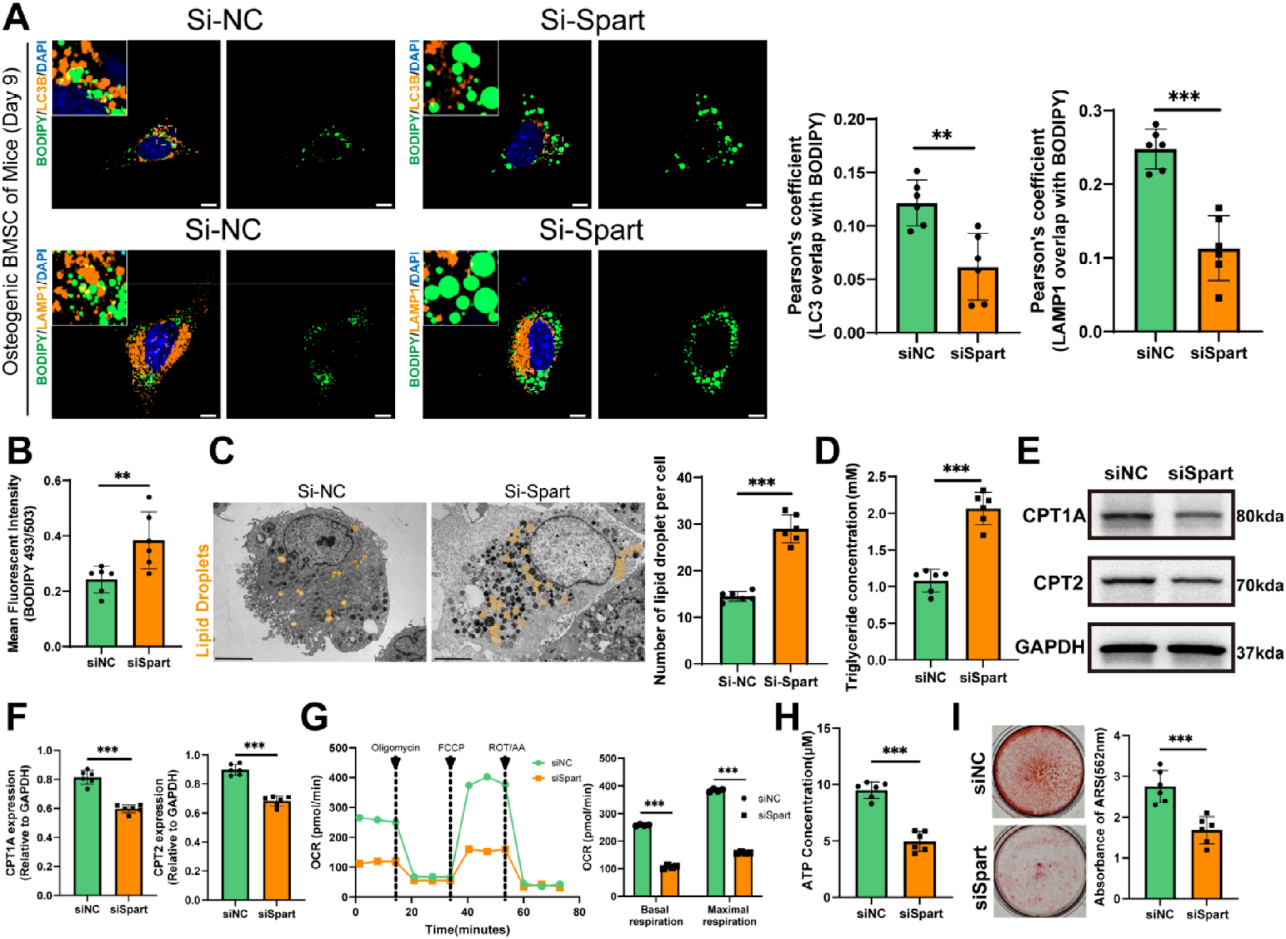
Inhibition of lipophagy compromises MSC osteogenic differentiation in vitro. (A) Upper: representative immunofluorescence micrographs of LC3 (orange) and BODIPY 493/503 (green) double‐staining to label autophagosomes and LDs after treated with siSpart; Lower: representative immunofluorescence micrographs of LAMP1 (orange) and BODIPY 493/503 (green) double‐staining to label autolysosomes and LDs after treated with siSpart. Results are presented as Pearson's coefficient(right). (scale bar = 10µm, *n =* 6). (B) Increased mean fluorescence intensity of BODIPY 493/503 (green) after treated with siSpart.(*n =* 6) (C) Representative TEM images showing increased intracellular lipid droplets (yellow) in MSCs after treated with siSpart, scale bar = 500nm. (*n =* 6) (D) Increased intracellular triglyceride content in MSCs after treated with siSpart. (*n =* 6) (E, F) Representative immunoblots (E) and quantitative analysis (F) showing decreased protein levels of the β‐oxidation key enzymes CPT1A and CPT2 in MSCs after treated with siSpart. (*n =* 6) (G) Curve and quantitative analysis of the OCRs for MSCs after treated with siSpart. (*n =* 4) (H) Quantification of decreased intracellular ATP levels in MSCs after treated with siSpart. (*n =* 4) (I) Representative Alizarin Red S staining and quantitative analysis reveal that siSpart treatment markedly attenuated the osteogenic mineralization capacity of MSCs. (*n =* 6). The Student's *t*‐test was used to analyze the differences between two groups, and the data are expressed as mean ± SD, *ns*: no significance; ^*^ p < 0.05, ^**^ p < 0.01, ^***^ p < 0.001.

Given that lipophagy is a continuous and selective form of autophagy, conventional autophagy inhibitors such as chloroquine and 3‐methyladenine (3‐MA) lack specificity, as they broadly suppress multiple types of selective autophagy and may therefore confound experimental interpretation. Recently, Jeeyun Chung et al. identified the Troyer‐syndrome protein SPARTIN (Spart) as a lipophagy‐specific receptor that mediates the recognition and degradation of lipid droplets. Knockdown of Spart markedly impairs lipophagic activity [19]. Building on this discovery, a similar approach was adopted to inhibit lipophagy in MSCs by silencing Spart expression. In this experiment, Spart expression was efficiently silenced in MSCs using siRNA (Si‐Spart) (Figure S2A,B). Immunofluorescence analysis revealed significantly reduced LC3B‐BODIPY and LAMP1–BODIPY colocalization in the Si‐Spart group, indicating attenuated lipophagic activity (Figure 2A). Consistently, lipid droplets accumulated markedly following Spart knockdown, with both the number and average volume of LDs increased (Figure 2B). These findings suggest that LDs accumulation resulted from impaired lipophagic degradation. Transmission electron microscopy further confirmed the pronounced LDs accumulation in Spart‐deficient MSCs (Figure 2C). Biochemical assays demonstrated that intracellular triglyceride levels were significantly elevated after Spart knockdown (Figure 2D), whereas free fatty acid (FFA) levels were markedly reduced (Figure S2C). At the level of FAO, protein expression of CPT1A and CPT2 was decreased in Spart‐deficient MSCs, reflecting an adaptive downregulation of mitochondrial fatty acid import in response to reduced availability of lipolytic substrates (Figure 2E,F). Consistent with these changes, Seahorse analysis showed that both basal and maximal respiration declined following Spart silencing (Figure 2G). ATP production via oxidative phosphorylation was significantly reduced (Figure S2D), and total intracellular ATP content was also lower in the Si‐Spart group (Figure 2H). Functionally, Alizarin Red S (ARS) staining, Alkaline Phosphatase (ALP) staining and activity assay, and Western blot analysis of the osteogenic transcription factors OCN and COL1A1 collectively revealed a substantial impairment in the osteogenic differentiation capacity of MSCs upon Spart knockdown (Figure 2I; Figure S2E,F). Collectively, these results demonstrate that suppression of Spart disrupts lipophagic flux in MSCs, leading to excessive LDs accumulation, diminished FAO, and reduced mitochondrial ATP generation. Consequently, under energy‐limited conditions, the highly energy‐demanding process of osteogenic differentiation is significantly compromised.

### 2.3 MSCs Derived from SOP Mice Exhibit Impaired Lipophagic Activity

**FIGURE 3:**
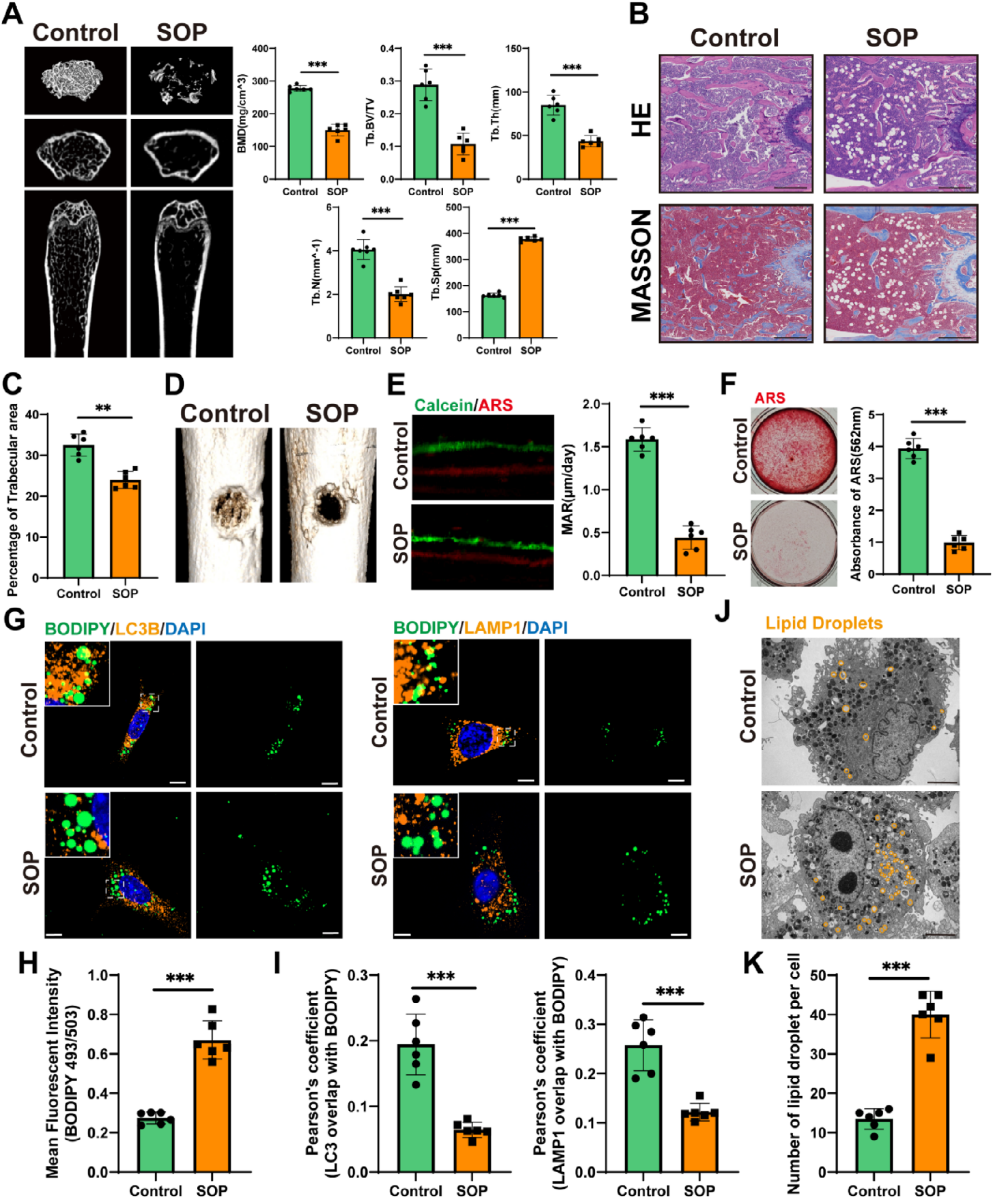
MSCs derived from SOP mice exhibit impaired lipophagic activity. (A) Representative micro‐CT images and quantification showing reduced bone mass and microarchitectural parameters, including BV/TV, Tb.Th, Tb.N, BMD and Tb.Sp, in SOP mice compared with controls (*n =* 6). (B, C) Representative histological images and quantification analysis of femurs stained with hematoxylin and eosin (H&E) and Masson's trichrome showing decreased trabecular bone and collagen deposition in SOP mice (scale bar = 200µm, *n =* 6). (D) Micro‐CT analysis showing the femoral defects of SOP and controls. (E) Representative fluorescence images of Calcein/Alizarin Red S double labeling showing reduced mineral apposition rate (MAR) in SOP compared with controls (*n =* 6). (F) Representative Alizarin Red S staining and quantification of mineralized area confirming impaired osteogenic differentiation capacity of SOP‐MSCs (*n =* 6). (G) Representative immunofluorescence images of BODIPY (green) co‐stained with LC3B or LAMP1 showing decreased colocalization between lipid droplets and autophagosomes/lysosomes in SOP‐MSCs.(scale bar = 10µm, *n =* 6). (H, I) Quantification of mean fluorescence intensity of BODIPY 493/503 (green) and Pearson's coefficient of immunofluorescence images. (*n =* 6). (J, K) Representative TEM images and quantification of lipid droplet number per cell showing the accumulation of undegraded lipid droplets (yellow) in SOP‐MSCs, and quantification of lipid droplet number per cell, scale bar = 500nm. (*n =* 6). The Student's *t*‐test was used to analyze the differences between two groups, and the data are expressed as mean ± SD, *ns*: no significance; ^*^ p < 0.05, ^**^ p < 0.01, ^***^ p < 0.001.

SOP is characterized by markedly reduced bone formation, and the impaired osteogenic differentiation capacity of MSCs represents a central pathogenic mechanism of this condition. However, whether MSCs exhibit lipophagic dysfunction in SOP remains unclear. To address this question, a senile osteoporosis mouse model was established using 22 – month—old C57BL/6J mice, while 3 – month—old mice were used as young controls. Micro‐CT scanning and 3D reconstruction of femoral trabeculae revealed severe bone mass loss and deterioration of trabecular microarchitecture in SOP mice compared with young controls (Figure 3A). Histological staining, including H&E and Masson's trichrome, further confirmed a significant reduction in trabecular bone area and a marked increase in marrow adiposity, consistent with typical skeletal aging phenotypes (Figure 3B,C). In addition, femoral drill‐hole repair assays demonstrated substantially delayed bone‐defect healing in SOP mice relative to controls (Figure 3D). Calcein double labeling showed that the mineral apposition rate in SOP mice was significantly lower than that of young controls (Figure 3E). Collectively, these findings indicate impaired bone formation and delayed mineralization in SOP mice.

In the subsequent phase, MSCs were isolated from both SOP and control mice, followed by the induction of osteogenic differentiation in vitro conditions. ARS staining, ALP staining, and activity assay, and Western blot analysis of the osteogenic transcription factors OCN and COL1A1 collectively revealed that SOP‐MSCs exhibited markedly reduced osteogenic mineralization compared with controls (Figure 3F; Figure S3H,I). To conduct a more in—depth assessment of whether lipophagy was impaired in SOP—MSCs, immunofluorescence staining for LC3, LAMP1, and BODIPY was carried out on day 9 of osteogenic differentiation. Relative to control MSCs, SOP‐MSCs contained substantially more intracellular LDs, whereas LC3‐BODIPY and LAMP1‐BODIPY colocalization was significantly decreased, indicating attenuated lipophagic flux and consequent lipid accumulation (Figure 3G–K). Transmission electron microscopy confirmed these observations, revealing increased LDs deposition in SOP‐MSCs. Biochemical assays showed that SOP‐MSCs displayed significantly lower intracellular FFA levels and higher triglyceride concentrations than control MSCs (Figure S3A,B). Western blot analysis further demonstrated reduced expression of CPT1A and CPT2 in SOP‐MSCs (Figure S3C). Consistent with these metabolic alterations, Seahorse analysis revealed markedly decreased basal and maximal oxygen consumption rates, along with diminished ATP production through oxidative phosphorylation (Figure S3D–F). Total intracellular ATP content was likewise reduced in SOP‐MSCs compared with controls (Figure S3G). Collectively, these findings demonstrate that in a murine model of senile osteoporosis, MSCs exhibit impaired lipophagic activity accompanied by reduced FAO‐OXPHOS‐driven ATP production, providing a mechanistic link between defective lipid catabolism and the diminished osteogenic potential characteristic of aged bone.

### 2.4 Specific Inhibition of Lipophagy in MSCs In Vivo Induces an Osteoporosis‐Like Phenotype in Mice

**FIGURE 4:**
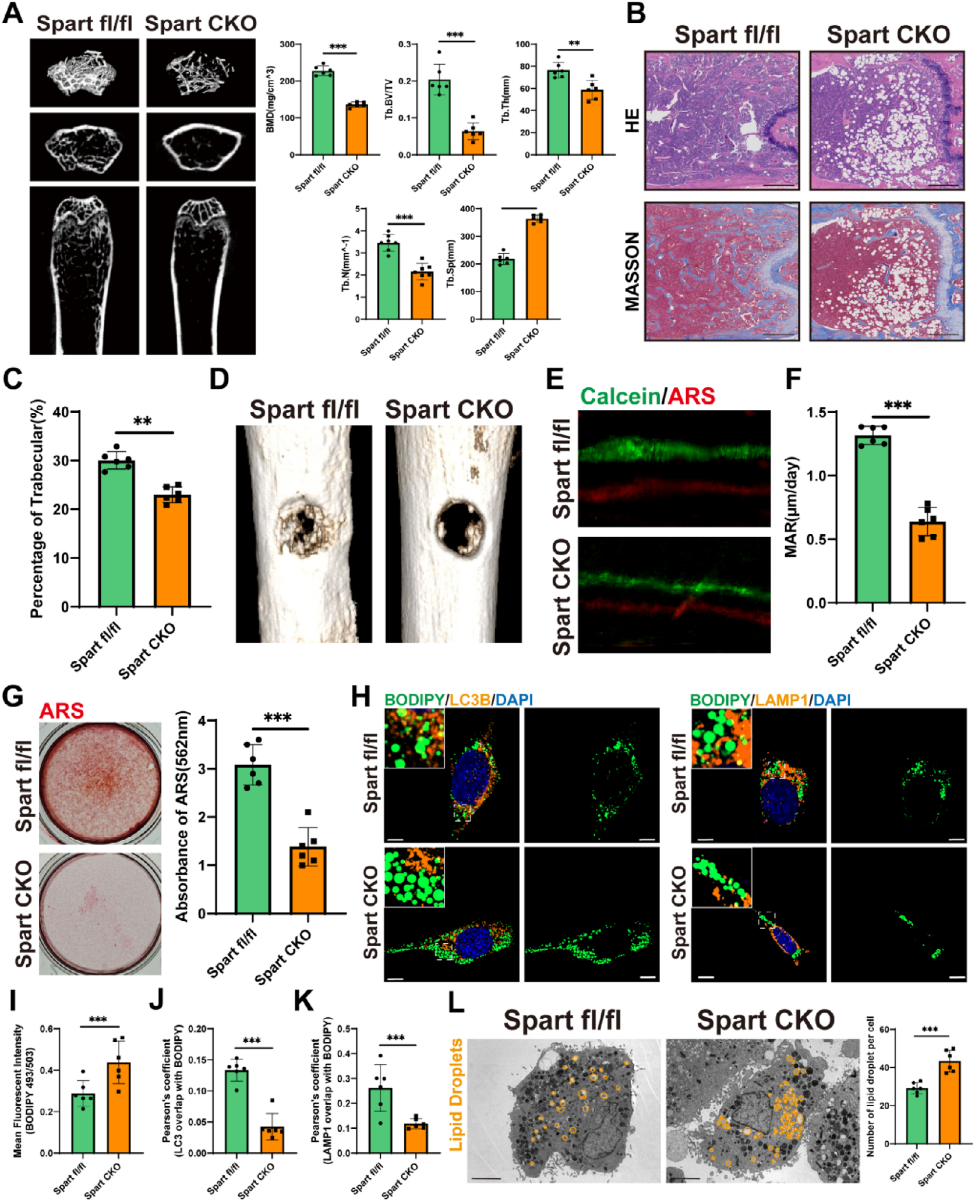
Specific inhibition of lipophagy in MSCs in vivo induces an osteoporosis‐like phenotype in mice. (A) Representative micro‐CT images and quantitative bone morphometric parameters showing reduced bone mass and trabecular microarchitecture (BV/TV, Tb.Th, Tb.N, Tb.Sp, BMD) in Spart CKO mice compared with Spart fl/fl controls (*n =* 6). (B, C) Representative histological images (H&E and Masson's trichrome) and quantification analysis of femoral sections showing decreased trabecular bone area and collagen deposition in Spart CKO mice (scale bar = 200µm, *n =* 6). (D) Representative femoral drill‐hole repair assay showing delayed bone regeneration in Spart CKO mice. (E, F) Representative Calcein/Alizarin Red S double labeling and quantification of mineral apposition rate (MAR) showing slower bone formation in Spart CKO mice (*n =* 6). (G) Representative Alizarin Red S staining of MSCs after 9 days of osteogenic induction showing markedly reduced mineralized nodule formation in Spart CKO MSCs compared with Spart fl/fl controls (*n =* 6). (H) Representative immunofluorescence images of BODIPY (green) co‐stained with LC3B or LAMP1. Spart CKO MSCs show markedly decreased colocalization of LC3B/LAMP1 with lipid droplets compared with controls, and (I–K) quantification of colocalization presented as Pearson's correlation coefficient (scale bar = 10µm, *n =* 6). (L) Representative TEM images and quantification of lipid droplet number per cell showing extensive LD accumulation in Spart CKO MSCs, scale bar = 500nm. (*n =* 6). The Student's *t*‐test was used to analyze the differences between two groups, and the data are expressed as mean ± SD, *ns*: no significance; ^*^ p < 0.05, ^**^ p < 0.01, ^***^ p < 0.001.

Although our in vitro findings demonstrated that MSCs rely on lipophagy‐derived energy during the late stage of osteogenic differentiation, and our SOP mouse model revealed impaired MSC lipophagic function, the in vivo environment is inherently complex, with multiple regulatory pathways influencing MSC‐mediated osteogenesis. Thus, whether lipophagy plays a causative role in the impaired bone formation associated with SOP remains unclear. To address this question, we generated a mouse model with MSC‐specific deletion of Spart, the lipophagy receptor, to disrupt lipophagic activity in vivo. Using CRISPR‐Cas9 genome editing, the Spart fl/fl mice were first generated and subsequently crossed them with Prx1‐CreERT2 mice to produce Spart fl/fl;Prx1‐CreERT2 conditional knockout mice (hereafter referred to as Spart CKO), in which SPARTIN is specifically ablated in MSCs (Figure S4A–C). As in the analyses performed in SOP mice, bone mass in Spart CKO mice was assessed by Micro‐CT. Micro‐CT scanning and 3D reconstruction of femoral trabeculae revealed a significant reduction in bone mass in 12‐month‐old Spart CKO mice compared with Spart fl/fl littermate controls (Figure 4A). Histological analyses using H&E and Masson's trichrome staining further confirmed a marked decrease in trabecular bone area in Spart CKO mice (Figure 4B,C). Functionally, femoral drill‐hole repair assays demonstrated substantially impaired bone‐defect healing in Spart CKO mice relative to controls (Figure 4D). Calcein double labeling revealed a markedly reduced mineral apposition rate in the femurs of Spart CKO mice (Figure 4E,F). Moreover, in vitro osteogenic induction of MSCs isolated from these mice showed that Spart CKO MSCs displayed significantly reduced mineralized nodule formation compared with Spart fl/fl MSCs, as evidenced by ARS staining, ALP staining, and activity assay, and Western blot analysis of the osteogenic transcription factors OCN and COL1A1 on day 9 of differentiation (Figure 4G; Figure S4K,L). Collectively, these results demonstrate that conditional deletion of Spart in MSCs leads to pronounced bone loss and diminished osteogenic capacity, resulting in an osteoporosis‐like phenotype in 12‐month‐old mice that closely mirrors the skeletal defects observed in 22‐month‐old SOP mice.

At the next stage, lipophagic activity in MSCs isolated from Spart fl/fl and Spart CKO mice was evaluated. Immunofluorescence staining for LC3B‐BODIPY and LAMP1‐BODIPY revealed a significant reduction in LC3B‐LDs and LAMP1‐LDs colocalization in Spart CKO MSCs compared with Spart fl/fl controls, indicating impaired lipophagic flux. In contrast, quantification of intracellular BODIPY fluorescence showed pronounced LDs accumulation in Spart CKO MSCs (Figure 4H–K). Transmission electron microscopy further confirmed these observations, demonstrating extensive LDs deposition in Spart CKO MSCs (Figure 4L). Together, these results indicate that MSC‐specific deletion of Spart markedly impairs lipophagic function in vivo. Biochemical analyses showed that intracellular FFA levels were significantly reduced, whereas triglyceride levels were elevated in Spart CKO MSCs (Figure S4D). The accumulated triglycerides could no longer be degraded via lipophagy to generate FFA (Figure S4E). Western blot analysis further revealed downregulation of the protein level of CPT1A and CPT2 (Figure S4F). Consistent with these metabolic alterations, Seahorse assays demonstrated reduced basal and maximal oxygen consumption rates in Spart CKO MSCs. ATP production through oxidative phosphorylation was markedly decreased, and total intracellular ATP content was significantly lower than in controls (Figure S4G–J). Collectively, these findings demonstrate that MSC‐specific ablation of Spart disrupts lipophagic degradation, leading to excessive lipid accumulation, reduced FAO and OXPHOS activity, and diminished ATP production. Consequently, these metabolic defects impair MSC osteogenic capacity, contributing to the development of an osteoporosis‐like phenotype at an earlier age.

### 2.5 Enhancing MSC Lipophagic Activity In Vivo Restores Bone Mass in both SOP and Spart CKO Mouse Models

**FIGURE 5:**
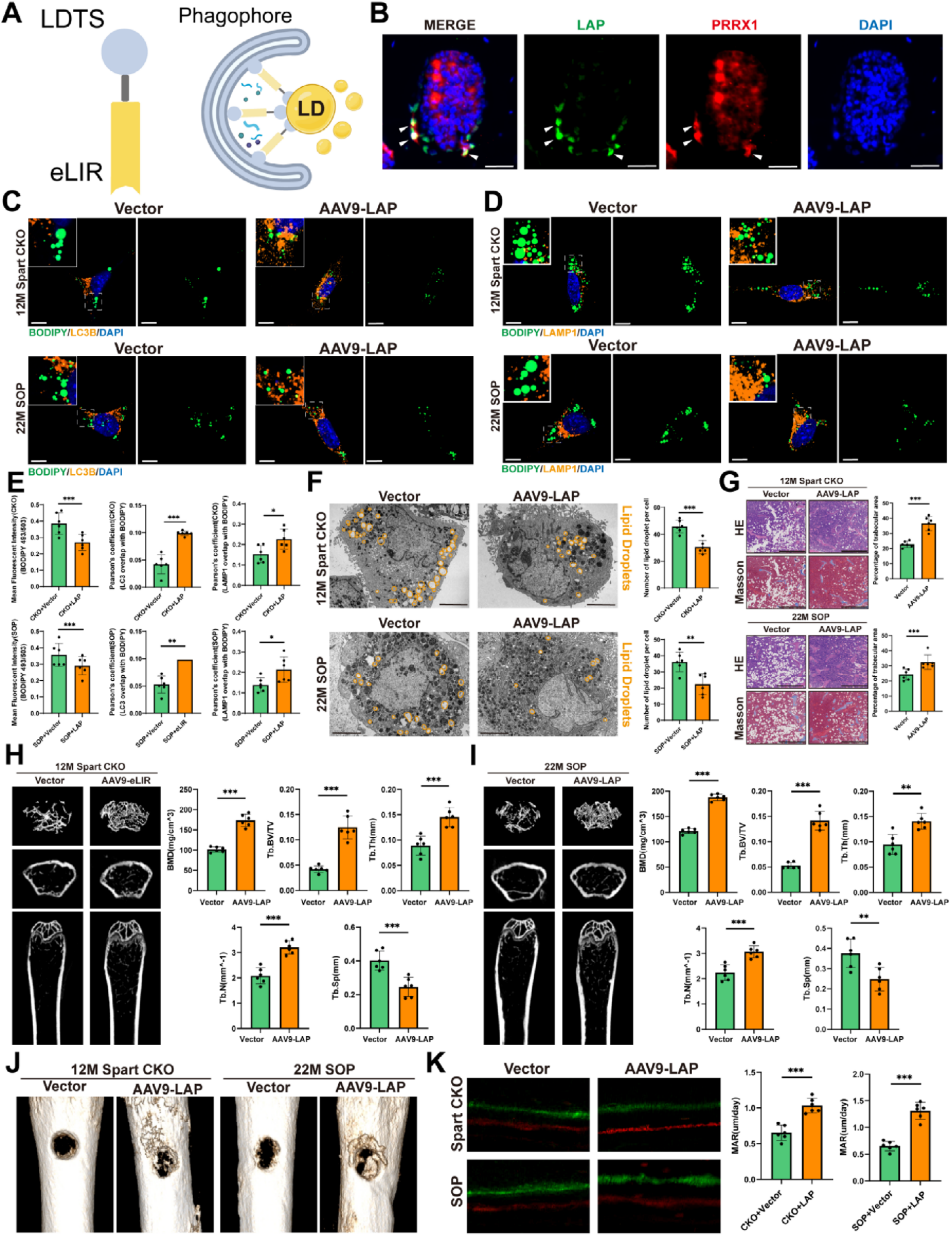
Enhancing MSC lipophagic activity in vivo restores bone mass in both SOP and Spart CKO mouse models. (A) Schematic illustration showing the structure of the lipophagy adaptor protein (LAP), consisting of an engineered LC3‐interacting region (eLIR) fused with a lipid‐droplet‐targeting signal (LDTS), designed to promote lipophagy. (B) Immunofluorescence images of bone sections showing colocalization of LAP (green) with PRRX1^+^ MSCs (red) after btAAV9‐LAP delivery. (scale bar = 200µm)(C, D) Representative immunofluorescence images of BODIPY (green) co‐stained with LC3B or LAMP1 in MSCs isolated from 12‐month Spart CKO and 22‐month SOP mice treated with vector or btAAV9‐LAP. Colocalization of LC3B/LAMP1 with lipid droplets was markedly increased after btAAV9‐LAP treatment. (scale bar = 10µm)(E) Quantitative analysis of BODIPY fluorescence intensity and Pearson's correlation coefficients for LC3B/LAMP1 colocalization with lipid droplets (*n =* 6). (F) Representative TEM images and quantification showing reduced intracellular lipid‐droplet accumulation in MSCs from btAAV9‐LAP–treated mice compared with vector controls, scale bar = 500 nm.(*n =* 6). (G) Representative histological images (H&E and Masson's trichrome) and quantitative analysis of femoral sections showing increased trabecular bone area and collagen deposition after btAAV9‐LAP treatment (scale bar = 200 µm, *n =* 6). (H, I) Representative micro‐CT images and quantitative bone morphometric parameters (BV/TV, Tb.Th, Tb.N, Tb.Sp, BMD) showing enhanced bone mass and trabecular architecture in btAAV9‐LAP–treated 12‐month Spart CKO and 22‐month SOP mice (*n =* 6) (J). Representative femoral defects‐repair assay showing improved bone regeneration following btAAV9‐LAP treatment. (K) Representative Calcein/Alizarin Red S double labeling and quantification of mineral apposition rate (MAR) demonstrating accelerated bone formation after btAAV9‐LAP treatment (*n =* 6). The Student's *t*‐test was used to analyze the differences between two groups, and the data are expressed as mean ± SD. *ns*: no significance; ^*^ p < 0.05, ^**^ p < 0.01, ^***^ p < 0.001.

Having established the essential role of MSC lipophagy in osteogenesis and demonstrated that lipophagic dysfunction contributes to the pathogenesis of SOP, it was investigated whether enhancing lipophagy in MSCs could restore their bioenergetic and osteogenic capacity and thereby improve bone mass. Inspired by the work of Yoshito Minami et al., a lipophagy adaptor protein (LAP) was generated to promote lipophagic activity [20]. This LAP consists of an engineered LC3‐interacting region (eLIR) fused to a lipid‐droplet–targeting signal (LDTS), enabling efficient initiation of lipophagy (Figure 5A). To specifically deliver LAP to bone‐resident MSCs, an adeno‐associated virus (AAV) vector was developed. Although recombinant AAV9 (rAAV9) can effectively target osteogenic cells in vivo, it retains high tropism for non‐skeletal organs such as the myocardium, skeletal muscle, alveoli, and liver. Previous work by Y. S. Yang et al. demonstrated that grafting a bone‐targeting peptide motif, (Asp‐Ser‐Ser)_6_, onto the VP2 capsid of AAV9 markedly enhances its affinity for osteogenic‐lineage cells while reducing off‐target accumulation [21]. Following this strategy, a bone‐tropic AAV9 vector carrying the LAP construct (btAAV9‐LAP) was successfully engineered, thereby facilitating efficient targeting of bone MSCs (Figure 5B).

12‐month‐old Spart CKO mice and 22‐month‐old SOP mice were intravenously administered btAAV9‐LAP and subsequently assessed MSC lipophagic activity in treated and untreated animals (Figure 5C,D). Immunofluorescence analysis revealed that LC3B–BODIPY colocalization was significantly increased in btAAV9‐LAP‐treated MSCs, accompanied by reduced LDs accumulation (Figure 5E). TEM analysis confirmed a pronounced reduction in intracellular LDs in MSCs from both Spart CKO and SOP mice after btAAV9‐LAP treatment compared with controls (Figure 5F). Biochemical measurements showed markedly reduced intracellular triglyceride levels and significantly elevated FFA levels in the btAAV9‐LAP group (Figure S5A,B), indicating that LAP enhanced LDs degradation via lipophagy. Western blot analysis further demonstrated partial restoration of the protein level of CPT1A and CPT2 in treated MSCs (Figure S5C). Consistent with these improvements, Seahorse analysis revealed increased basal and maximal respiration in the btAAV9‐LAP group, accompanied by enhanced ATP production through oxidative phosphorylation and higher total intracellular ATP levels compared with untreated controls (Figure S5D–J). Collectively, these findings indicate that targeted expression of LAP in bone‐resident MSCs enhances lipophagic flux, restores mitochondrial energy metabolism, and reactivates osteogenic potential in both Spart CKO and SOP mice.

### 2.6 Digoxin Restores MSC Lipophagic Activity and Bone Mass in SOP Mice but Induces Cardiac Side Effects

**FIGURE 6:**
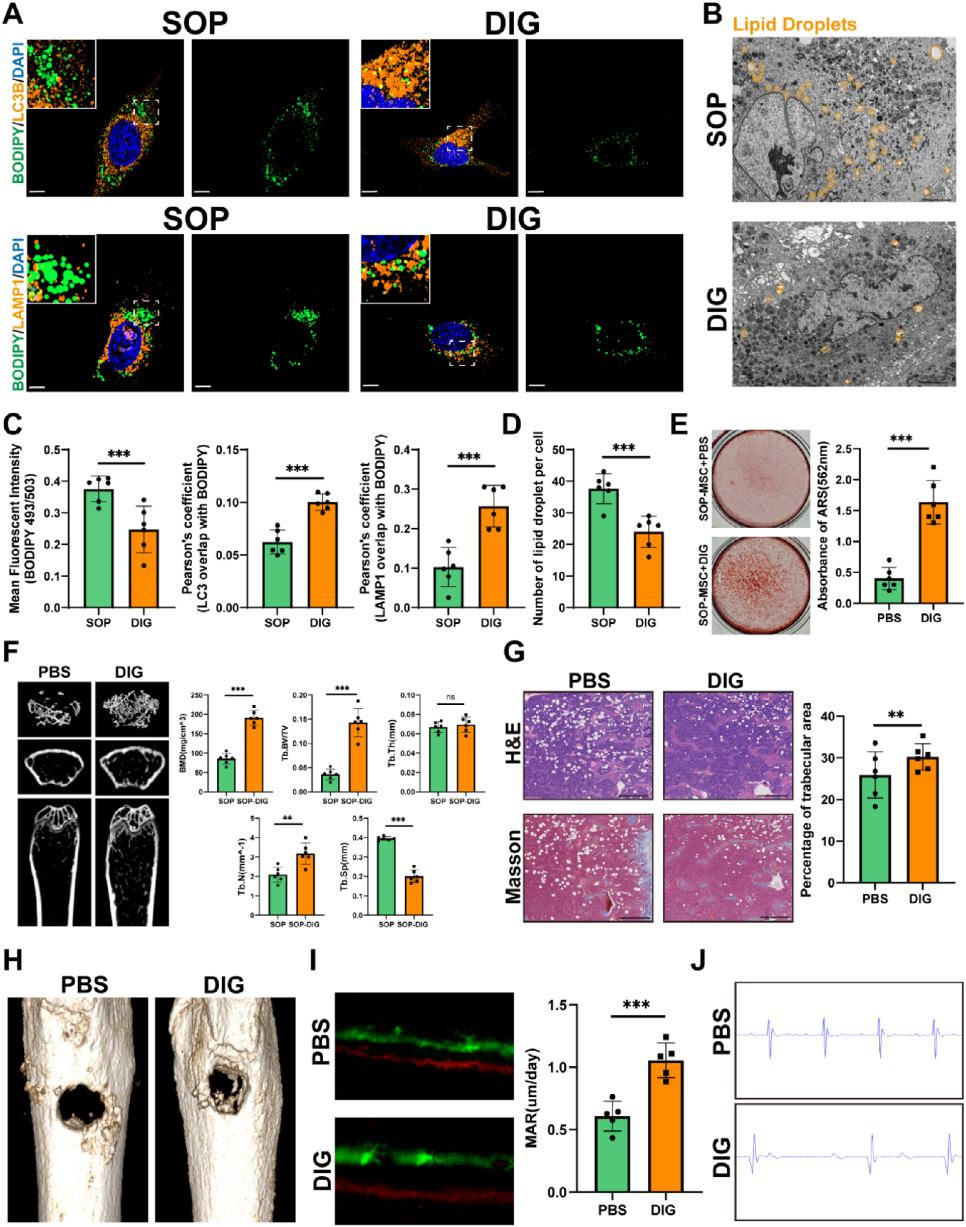
Digoxin restores MSC lipophagic activity and bone mass in SOP mice but induces cardiac side effects. (A) Representative immunofluorescence images of MSCs isolated from 22‐month SOP mice showing colocalization of BODIPY (green) with LC3B or LAMP1 after digoxin (DIG) treatment. Digoxin markedly increased LC3B/LAMP1 colocalization with lipid droplets compared with SOP controls (scale bar = 10µm, *n =* 6). (B) Representative TEM images showing reduced intracellular lipid‐droplet (yellow) accumulation in MSCs from DIG‐treated SOP mice, scale bar = 500 nm. (*n =* 6). (C) Quantification of mean BODIPY fluorescence intensity and Pearson's correlation coefficients for LC3B and LAMP1 colocalization with BODIPY (*n =* 6). (D) Quantification of lipid‐droplet number per cell showing significant reduction after DIG treatment (*n =* 6). (E) Representative Alizarin Red S staining of MSCs after osteogenic induction showing enhanced mineralized nodule formation in DIG‐treated MSCs compared with PBS controls (*n =* 6). (F) Representative micro‐CT images and quantitative bone morphometric parameters (BV/TV, Tb.Th, Tb.N, Tb.Sp, BMD) showing increased bone mass and improved trabecular microarchitecture in DIG‐treated SOP mice (*n =* 6). (G) Representative histological images (H&E and Masson's trichrome) and quantitative analysis showing enlarged trabecular bone area after DIG treatment (scale bar = 200 µm, *n =* 6). (H) Representative femoral drill‐hole repair assay showing accelerated bone‐defect healing in DIG‐treated mice. (I) Representative Calcein/Alizarin Red S double labeling and quantification of mineral apposition rate (MAR) showing faster calcium‐salt deposition in DIG‐treated SOP mice (*n =* 6). (J) Representative ECG recordings showing cardiac arrhythmia in DIG‐treated mice, indicating dose‐dependent cardiotoxicity. The Student's *t*‐test was used to analyze the differences between two groups, and the data are expressed as mean ± SD. *ns*: no significance; ^*^ p < 0.05, ^**^ p < 0.01, ^***^ p < 0.001.

Digoxin (DIG) has previously been reported to enhance cellular lipophagic activity. Clinical evidence further shows that among elderly individuals (>65 years old) with cardiac arrhythmia, digoxin use is associated with a dose‐dependent reduction in the incidence of SOP‐related hip and forearm fractures, whereas other antiarrhythmic agents exhibit no comparable effect [22]. These observations strongly suggest a potential therapeutic role for digoxin in SOP and osteoporotic fracture prevention. To further assess the relationship between digoxin use and bone mineral density (BMD), the NHANES database (2005‐2018) was analysed. A total of 201 digoxin users aged >65 years were included, along with 367 age‐, sex‐, and race‐matched nonusers as controls. Two multivariate linear regression models were constructed: Model 1 adjusted for age, race, and sex, and Model 2 additionally adjusted for smoking status, BMI, chronic kidney disease (CKD), and fracture history. In Model 1, digoxin use was significantly and positively associated with total and lumbar (L1‐L4) BMD, whereas BMD of the total femur, femoral neck, trochanter, intertrochanteric region, and Ward's triangle showed mild but nonsignificant positive trends (Figure S6A). After additional covariate adjustment in Model 2, digoxin use remained significantly associated with total and lumbar BMD (Figure S6B), while the positive but nonsignificant associations with femoral BMD indices persisted. These results indicate that digoxin administration may contribute to higher BMD in elderly individuals older than 65 years. The therapeutic potential of digoxin in the SOP mouse model was evaluated. MSCs isolated from digoxin‐treated SOP mice displayed markedly enhanced lipophagic activity, as evidenced by increased LC3B–BODIPY and LAMP1–BODIPY colocalization and reduced intracellular LDs accumulation (Figure 6A,C). Transmission electron microscopy corroborated these findings by revealing fewer intracellular LDs in digoxin‐treated MSCs compared with untreated SOP MSCs (Figure 6B,D). Biochemical analyses further demonstrated decreased intracellular triglyceride levels and increased FFA levels following digoxin treatment (Figure S6C,D). Western blot analysis showed elevated expression of the protein level of CPT1A and CPT2 in MSCs from digoxin‐treated SOP mice (Figure S6E). Consistent with these metabolic improvements, Seahorse analysis revealed higher basal and maximal oxygen consumption rates in MSCs from the digoxin‐treated group (Figure S6F). ATP production through oxidative phosphorylation was restored after digoxin treatment (Figure S6G), accompanied by a parallel increase in total intracellular ATP content. Functionally, ARS staining, ALP staining, and activity assay, and Western blot analysis of the osteogenic transcription factors OCN and COL1A1 collectively confirmed that digoxin treatment rescued the impaired osteogenic differentiation capacity of SOP‐derived MSCs (Figure 6E; Figure S6I,J). Collectively, these findings demonstrate that digoxin effectively restores lipophagic activity and mitochondrial energy metabolism in MSCs from SOP mice, thereby rescuing their osteogenic potential.

To evaluate the effects of digoxin on bone mass, femoral micro‐CT scanning was performed in SOP mice. Digoxin‐treated mice exhibited a significant increase in bone mass compared with controls, with partial restoration of trabecular parameters, including BMD, BV/TV, Tb.Th, Tb.Sp, and Tb.N (Figure S6K; Figure 6F). Histological examination of femoral sections further confirmed a marked increase in trabecular area following digoxin treatment, as demonstrated by H&E and Masson's trichrome staining (Figure 6G). Functionally, femoral drill‐hole repair assays showed that digoxin administration accelerated bone‐defect healing, resulting in smaller residual defects than those observed in untreated SOP mice (Figure 6H). Similarly, calcein double‐labeling analysis revealed faster calcium deposition rates after digoxin treatment (Figure 6I). Despite these beneficial effects on MSC lipophagy and osteogenesis, high‐dose digoxin (0.8 mg/kg)—the dose required to achieve therapeutic efficacy against SOP—induced notable cardiac arrhythmias (Figure 6J). Histopathological analysis of cardiac tissue revealed localized myocardial necrosis in digoxin‐treated mice (Figure S6L), indicating significant cardiotoxicity. Collectively, these findings demonstrate that although digoxin effectively enhances MSC lipophagy, restores bone mass, and ameliorates osteoporotic bone loss in SOP mice, its clinical utility is severely constrained by dose‐dependent cardiotoxic side effects. Thus, improving the tissue specificity and safety profile of digoxin delivery is essential for its translational potential in osteoporosis therapy.

### 2.7 SNM@NP‐DIG Targets Bone Marrow to Enhance MSC Lipophagy and Alleviate Osteoporosis in SOP Mice

**FIGURE 7:**
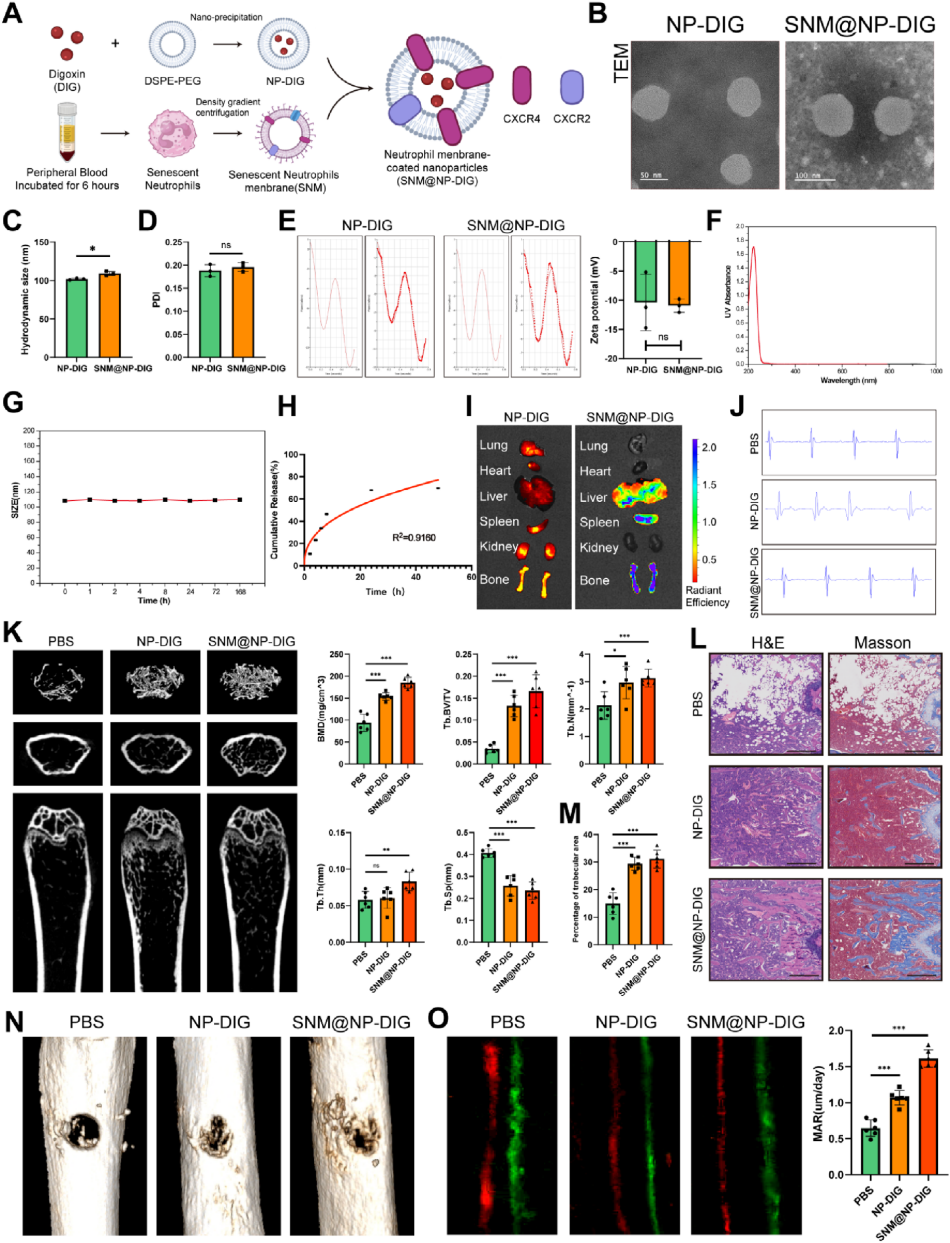
SNM@NP‐DIG targets bone marrow to enhance MSC lipophagy and alleviate osteoporosis in SOP mice. (A) Schematic illustration of the preparation process for senescent neutrophil membrane–coated digoxin‐loaded nanoparticles (SNM@NP‐DIG). Peripheral blood–derived neutrophils were incubated for 6 h to induce senescence, and their membranes were isolated and used to coat PEG‐based digoxin nanoparticles (NP‐DIG). The high CXCR4 expression on senescent neutrophil membranes enabled bone marrow homing and enhanced targeting capability. (B) Representative TEM images showing the spherical morphology and uniform particle size of NP‐DIG and SNM@NP‐DIG. (C– F) Quantification of hydrodynamic size, PDI, and zeta potential showing slightly increased particle diameter of SNM@NP‐DIG (50–150 nm range) compared with NP‐DIG, with stable dispersion and favorable biocompatibility. UV–vis absorbance spectra confirmed successful fabrication of SNM@NP‐DIG. (G–H) Stability and cumulative release profile of SNM@NP‐DIG showing good size stability in serum over 7 days and a biphasic “burst–sustained” release pattern with ∼70% cumulative release within 30 h. The cumulative release data were fitted to the Korsmeyer‐Peppas model (Q = k·t^n^, where Q is the cumulative drug release (%) and t is time (h)). The fitting yielded a release exponent (n) of 0.4071 and a coefficient of determination (R^2^) of 0.9160. (I) Representative in vivo fluorescence imaging (Cy5.5 labeling) showing biodistribution of NP‐DIG and SNM@NP‐DIG in major organs. SNM@NP‐DIG exhibited enhanced accumulation in bone marrow and reduced distribution in the heart compared with NP‐DIG, confirming improved bone‐targeting efficiency. (J) Representative ECG recordings showing that SNM@NP‐DIG caused fewer arrhythmias than NP‐DIG, suggesting mitigated cardiotoxicity. (K) Representative micro‐CT images and quantitative bone morphometric parameters (BV/TV, Tb.Th, Tb.N, Tb.Sp, BMD) showing that both NP‐DIG and SNM@NP‐DIG restored bone mass, with SNM@NP‐DIG producing the strongest improvement (*n =* 6). (L) Representative histological images (H&E and Masson's trichrome) showing increased trabecular bone area and collagen deposition after SNM@NP‐DIG treatment compared with PBS control (scale bar = 200 µm, *n =* 6). (M) Quantitative analysis of trabecular morphometry confirming enhanced bone quality after SNM@NP‐DIG administration (*n =* 6). (N) Representative femoral drill‐hole repair assay showing accelerated bone‐defect healing in SNM@NP‐DIG–treated mice compared with PBS or NP‐DIG groups. (O) Representative Calcein/Alizarin Red S double labeling and quantification of mineral apposition rate (MAR) showing enhanced calcium deposition in SNM@NP‐DIG–treated mice (*n =* 6). The Student's *t*‐test was used to analyze the differences between two groups, and the data are expressed as mean ± SD. *ns*: no significance; ^*^ p < 0.05, ^**^ p < 0.01, ^***^ p < 0.001.

To achieve bone marrow‐targeted delivery of digoxin and minimize its cardiotoxic side effects, a strategy inspired by the work of Zhenyu Luo et al. was performed [23]. Specifically, polyethylene glycol (PEG) nanoparticles coated with membranes derived from senescent neutrophils (SNM) were employed as drug carriers. The high expression of CXCR4 receptors on senescent neutrophil membranes enables homing to bone marrow, thereby conferring intrinsic bone‐targeting capability (Figure S7A). The construction of SNM@NP‐DIG is illustrated schematically (Figure 7A). Transmission electron microscopy revealed that SNM@NP‐DIG nanoparticles were uniformly spherical in morphology (Figure 7B). Hydrodynamic size analysis indicated that SNM@NP‐DIG exhibited a modest increase in diameter relative to NP‐DIG, remaining within the favorable 50–150 nm range conducive to enhanced biocompatibility and targeted delivery (Figure 7C). Further characterization demonstrated a narrow particle size distribution, as evidenced by low polydispersity index (PDI) values, and stable surface potential as measured by zeta potential analysis, both confirming satisfactory uniformity and physicochemical stability (Figure 7D,E). UV–vis absorbance spectra, in combination with TEM imaging, verified the successful synthesis of the desired nanocomposite (Figure 7F). Next the colloidal stability of SNM@NP‐DIG in serum was evaluated. Dynamic light scattering showed that the nanoparticle size remained stable over a one‐week incubation period, indicating excellent dispersion stability (Figure 7G). Cumulative release studies revealed a biphasic ‘burst–sustained’ release profile, with approximately 70% of digoxin released within 30 h (Figure 7H).

The in vivo biodistribution and therapeutic performance of SNM@NP‐DIG were evaluated. Cy5.5 fluorescence labeling revealed that SNM@NP‐DIG exhibited markedly enhanced bone‐targeting capability compared with uncoated NP‐DIG nanoparticles, as evidenced by stronger fluorescent accumulation in the skeletal regions (Figure 7I). Although moderate uptake was still observed in the liver and spleen, cardiac distribution of the drug was substantially reduced. Electrocardiographic analysis showed that while NP‐DIG treatment induced varying degrees of atrioventricular conduction block, mice receiving SNM@NP‐DIG displayed a markedly lower incidence of arrhythmia (Figure 7J). Consistently, histological examination of cardiac sections revealed significantly reduced myocardial injury in the SNM@NP‐DIG group relative to NP‐DIG‐treated mice (Figure S7B). Micro‐CT analysis demonstrated that SNM@NP‐DIG treatment effectively restored bone mass in SOP mice (Figure 7K). Histological staining further confirmed that the trabecular bone area was substantially increased in SNM@NP‐DIG–treated mice compared with the PBS control group (Figure 7L,M). Functional assays corroborated these findings: femoral drill‐hole repair experiments revealed that SNM@NP‐DIG promoted bone‐defect healing to a degree comparable to NP‐DIG treatment (Figure 7N), and calcein double labeling demonstrated accelerated calcium deposition following SNM@NP‐DIG administration (Figure 7O).

Together, these results indicate that SNM@NP‐DIG confers superior therapeutic efficacy compared with conventional NP‐DIG, effectively promoting bone regeneration while substantially mitigating digoxin‐induced cardiotoxicity through bone‐marrow‐targeted delivery.

## 3 Discussion

Elizabeth Rendina‐Ruedy and her colleagues have convincingly demonstrated that abundant lipid droplet accumulation occurs during the osteogenic differentiation of MSCs [12, 24]. This process triggers the activation of intrinsic lipolytic pathways, whereby sequential events of lipid droplet hydrolysis, oxidative phosphorylation, and ATP generation collectively fulfill the bioenergetic demands essential for MSC osteogenesis and matrix mineralization. Earlier investigations into the metabolic basis of MSC differentiation predominantly emphasized glucose‐derived energy metabolism, whereas the contribution of lipid‐driven bioenergetics has only recently garnered attention [25, 26]. Notably, FAO is markedly more efficient than glucose oxidation in producing ATP‐the complete oxidation of a single palmitate molecule yields nearly four times more ATP than that of glucose. Given that osteogenic differentiation and mineralization are highly energy‐intensive processes, FAO likely represents a pivotal metabolic source sustaining these events [27, 28]. Subsequent studies have provided substantial evidence supporting this hypothesis, as Rendina‐Ruedy and colleagues further demonstrated that endogenous classical lipolysis constitutes a major source of fatty acids during MSC osteogenesis [12].

Recent research has revealed that, beyond canonical lipolysis, a selective form of autophagy termed lipophagy can also degrade intracellular lipid droplets, liberating fatty acids to fuel mitochondrial FAO‐OXPHOS and ATP production [29, 30, 31]. However, whether lipophagy contributes to osteogenesis and mineralization in MSCs has remained elusive. This study provides the first evidence that endogenous lipophagy is activated during MSC osteogenic differentiation, promoting the hydrolysis of lipid droplets accumulated within differentiating MSCs. The liberated fatty acids subsequently fuel ATP generation through the FAO‐OXPHOS axis. Inhibition of endogenous lipophagy markedly suppressed ATP production and impaired osteogenic differentiation, underscoring its indispensable bioenergetic role. Collectively, these findings identify endogenous lipophagy as a previously unrecognized source of intracellular fatty acids and ATP during MSC osteogenesis, highlighting its critical regulatory function in bone homeostasis. This work thus opens a new avenue for investigating the intricate interplay between energy metabolism and osteogenic mineralization.

Insufficient osteogenesis mediated by SOP‐MSCs represents a key pathogenic mechanism underlying age‐related osteoporosis. Previous studies have shown that defective mitochondrial ATP generation is a major contributor to impaired osteogenic mineralization in SOP‐MSCs [32, 33]; however, the cause of this bioenergetic deficiency remains poorly defined. Autophagy has been recognized as essential for MSC osteogenic differentiation [34, 35], yet SOP‐MSCs exhibit defective autophagic activity, and restoration of autophagy only partially rescues their osteogenic capacity [36]. The precise mechanisms linking autophagy dysfunction to impaired osteogenesis, therefore, remain obscure. This study identifies and validates, for the first time, that endogenous selective autophagy‐specifically, lipophagy‐is functionally impaired during the osteogenic process of SOP‐MSCs. Restoration of lipophagic activity markedly enhanced mitochondrial ATP production and consequently rescued the osteogenic potential of SOP‐MSCs, thereby ameliorating senescence‐associated osteoporosis. Collectively, these findings interconnect three defining hallmarks of SOP‐MSC senescence—lipid droplet accumulation, autophagic dysfunction, and insufficient ATP generation—into a unified mechanistic framework. This conceptual advance provides a novel basis for understanding the bioenergetic failure underlying defective osteogenesis in SOP‐MSCs and highlights lipophagy as a potential therapeutic target for senile osteoporosis.

Building upon these mechanistic insights, further exploration was conducted on the therapeutic potential of lipophagy activation in SOP‐MSCs. AAV vectors, characterized by low immunogenicity and long‐term transgene expression after a single administration, represent an ideal system for chronic conditions such as SOP [21]. Inspired by the work of Y. S. Yang and colleagues, our previous studies developed a btAAV9 vector capable of systemically delivering therapeutic genes such as Bmal1 or Ttk, which substantially improved osteogenesis and bone mass in aged osteoporotic mice [37]. In the current study, the strategy was expanded through the engineering of a btAAV9 vector encoding a lipophagy adaptor protein to specifically target SOP‐MSCs. This approach effectively reactivated lipophagic flux, restored mitochondrial ATP generation, enhanced osteogenic differentiation, and ultimately ameliorated SOP in vivo.

Digoxin, a well‐characterized pharmacological activator of lipophagy [20], was found in both in vitro and in vivo experiments to enhance ATP production in SOP‐MSCs by stimulating lipophagic activity. This metabolic reactivation promoted osteogenesis and alleviated senile osteoporosis. However, due to digoxin's narrow therapeutic window—where effective and toxic doses are closely aligned‐its clinical application is limited by a substantial risk of cardiotoxicity, necessitating stringent dosage control. Animal studies further substantiated this concern, indicating that conventional digoxin administration may not be clinically feasible for SOP treatment. To enhance the bone‐targeting efficacy and simultaneously minimize systemic toxicity, nanomaterial‐based delivery strategies were incorporated. Neutrophils, which can be readily isolated from peripheral blood and are autologous in origin, offer an immune‐compatible vehicle for drug delivery. Notably, senescent neutrophils display high surface CXCR4 expression, conferring intrinsic bone marrow‐homing capability [23, 38, 39]. PEG nanoparticles, renowned for their excellent biocompatibility, low immunogenicity, and prolonged systemic circulation, are widely used in clinical drug delivery applications and have been approved by the U.S. Food and Drug Administration (FDA) [40]. Accordingly, a digoxin‐loaded delivery system was formulated, wherein PEG nanoparticles were enveloped with membranes sourced from senescent neutrophils. In vivo experiments demonstrated that these biomimetic nanoparticles exhibited excellent bone marrow‐targeting capacity, effectively enhancing the lipophagy‐activating function of digoxin while markedly reducing its cardiotoxicity in mice.

While digoxin has shown promise as a lipophagy activator in the present study, its potential direct clinical application may be substantially constrained by several well‐recognized limitations, including a narrow therapeutic window, marked inter‐individual pharmacokinetic variability driven primarily by renal function decline, and significant drug‐drug interactions. These concerns may be particularly pronounced in elderly osteoporosis patients, who frequently exhibit reduced renal reserve and require polypharmacy, potentially rendering them more susceptible to digoxin accumulation and toxicity. We acknowledge that further clinical pharmacological studies would be needed to fully characterize these risks in this patient population.

Compared with existing standard‐of‐care regimens for senile osteoporosis, including antiresorptive agents (bisphosphonates, denosumab) and anabolic agents (teriparatide, romosozumab), the lipophagy‐targeting strategy explored in the present study appears to address a distinct mechanism: the intrinsic metabolic dysfunction of MSCs. Current therapies primarily modulate systemic osteoclast‐osteoblast balance, and whether they sufficiently correct the impaired intracellular energy metabolism underlying age‐related decline in MSC osteogenic potential remains an open question. By attempting to restore SPARTIN‐mediated lipophagy and the downstream FAO‐OXPHOS axis, this approach may target a contributing metabolic mechanism of MSC osteogenic dysfunction. The bone‐targeted SNM@NP‐DIG delivery system was designed to further mitigate digoxin's clinical limitations by achieving preferential drug enrichment in the bone marrow niche while reducing systemic exposure, which may help to lower cardiotoxicity risk and inter‐individual variability‐related adverse events, though direct clinical validation remains to be performed.

Taken together, the present study proposes two potentially promising strategies to restore lipophagic flux in SOP‐MSCs, which may offer a basis for future therapeutic development for senile osteoporosis. Further investigations will be needed to evaluate the clinical feasibility and translational potential of these lipophagy‐targeted approaches.

Despite the encouraging findings of this study, several important limitations should be acknowledged. First, although the present results suggest that enhancing lipophagy may improve metabolic energy production and osteogenic differentiation in aged MSCs, the long‐term safety profile and biodistribution of the nanoparticle‐based delivery system have not yet been evaluated in extended in vivo models, and such studies will be necessary before any translational application can be considered. Second, while a functional association between lipophagy, FAO–OXPHOS reprogramming, and osteogenesis has been established, the precise upstream regulatory mechanisms and signaling pathways involved remain incompletely understood and warrant further investigation. Third, the optimal intensity of lipophagy activation and its potential impact on oxidative stress and cellular homeostasis have not been fully characterized in the present study, and defining a safe and effective therapeutic window will require additional work.

Future studies should aim to optimize the targeted delivery efficiency and dosage control of the lipophagy‐modulating strategy, systematically evaluate its long‐term biosafety in more clinically relevant models, and validate therapeutic efficacy in large‐animal systems. In addition, a deeper exploration of the interaction between lipophagy‐mediated metabolic reprogramming and other key signaling pathways in bone metabolism may help to further advance the translational potential of this approach, though we recognize that considerable work remains before clinical application could be contemplated.

This study establishes a simple yet compelling concept: endogenous lipophagy is activated during MSC osteogenic differentiation, orchestrating a coordinated cascade that couples lipid droplet hydrolysis, fatty acid oxidation, oxidative phosphorylation, and ATP generation to sustain osteogenesis and matrix mineralization. Aging disrupts this intrinsic lipophagic machinery, impairing the lipolysis‐FAO‐OXPHOS axis and leading to insufficient ATP production, defective osteogenesis, and the onset of SOP. By restoring endogenous lipophagic flux within SOP‐MSCs, the results demonstrated a marked enhancement in mitochondrial bioenergetics, a rescue of osteogenic differentiation, and an effective alleviation of SOP in vivo. In summary, this work provides the first demonstration that endogenous lipophagy is an essential regulator of cellular bioenergetics during MSC osteogenesis. Targeted restoration of lipophagic flux in SOP‐MSCs thus represents a promising and clinically translatable therapeutic avenue for the treatment of age‐related osteoporosis and impaired bone formation.

## 4 Conclusions

In summary, our study identifies MSC lipophagy as a fundamental metabolic mechanism governing osteogenic differentiation and reveals its dysfunction as a key driver of skeletal aging in senile osteoporosis. Impaired lipophagic flux in SOP and Spart‐deficient mice led to excessive lipid‐droplet accumulation, reduced FFA availability, suppressed FAO and OXPHOS activity, and ultimately compromised MSCs energy supply and osteogenic potential. Enhancing lipophagy through targeted LAP expression effectively restored mitochondrial metabolism, promoted mineralization, and ameliorated osteoporosis‐like phenotypes. Although digoxin similarly reactivated lipophagy and improved bone formation, its therapeutic utility was restricted by dose‐dependent cardiotoxicity. Importantly, engineering a bone marrow–targeted nanodelivery system (SNM@NP‐DIG) markedly increased drug specificity, minimized cardiac injury, and achieved substantial bone regeneration in SOP mice. Together, these findings establish MSC lipophagy as a central metabolic regulator of bone homeostasis and highlight bone‐targeted lipophagy activation as a promising therapeutic strategy for senile osteoporosis.

## 5 Experimental Section

### 5.1 Study Approval

This study was approved by the Ethics Committee of the Eighth Affiliated Hospital, Sun Yat‐Sen University, Shenzhen, China.

### 5.2 Cell Isolation and Culture

After bone marrow punctures of volunteers, density gradient centrifugation at 12 000 r·min−1 for 30 min (Invitrogen) was used to extract MSCs from the bone marrow. The extracted MSCs were cultured in Dulbecco's modified Eagle's medium (DMEM, Gibco, Cat.) containing 10% fetal bovine serum (Procell system, Cat.164210).

For mouse MSC isolation, C57BL/6 mice were sacrificed by cervical dislocation, then disinfected and soaked in 75% ethanol for 5–10 min. Under aseptic conditions, isolate femurs and tibias, cut into pieces less than 3 mm^3^. Filter through a 40 µm cell strainer (Servicebio, Cat.G6088‐40), and the filtrate is treated with red blood cell lysis buffer (Solarbio, Cat.R1010). After centrifugation, resuspend cells in complete α‐MEM medium (α‐MEM(Gibco, Cat.32571036), 10% fetal bovine serum(Procell system, Cat.164210), 1% penicillin/streptomycin(Biosharp, Cat.BL505A)), and incubate in a humidified incubator (37°C, 5% CO_2_). When cells in the culture flask reached a certain density, they were counted and seeded at an appropriate density into cell cultural plates. Upon reaching 80% confluence, MSCs were transferred to osteogenic induction medium (containing complete α‐MEM, 50 µg·mL^−^^1^ ascorbic acid (Sigma; Cat.A4544), and 5 mmol·L^−^^1^ β‐glycerophosphate (Sigma; Cat.G9422)) for culture to induce their differentiation into osteoblasts. For control group experiments, osteogenic induction medium was not used, and cells were maintained in complete medium throughout the culture period.

### 5.3 siRNA Infection

SPARTIN siRNAs were purchased from GenePharma (Shanghai, China)

### 5.4 ARS Assay

MSCs were rinsed twice with phosphate‐buffered saline (PBS), and then, 4% paraformaldehyde (PFA) was used to fix MSCs for 30 min.

For ARS staining, MSCs were stained with 1% ARS (pH 4.2) (Solarbio, Cat. No. G8550) for 15 min at room temperature. After removal of the nonspecific stains with PBS, the images of stained MSCs were captured. Cetylpyridinium chloride monohydrate (10%, Sigma–Aldrich, Cat.8400080100) was used to extract ARS staining for quantification.

### 5.5 Animal Models

22‐months‐old wild‐type C57BL/6 mice were purchased from the Laboratory Animal Center of Sun Yat‐Sen University. The murine experiments were approved by the Institutional Animal Care and Use Committee of Sun Yat‐Sen University, Guangzhou, China.

### 5.6 SPARTIN fl/fl;Prx1Cre CKO Mice

C57BL/6 Prx1CreER mice were purchased from the Jackson Laboratory. C57BL/6 SPARTIN fl/fl transgenic mice were purchased from SHANGHAI MODEL ORGANISMS. The SPARTIN fl/fl; Prx1CreER mice were generated by crossing SPARTIN fl/fl mice with Prx1CreER mice. Tamoxifen was administered by intraperitoneal injection at a dose of 75 mg/kg/day for one week to these mice at the 12th month after birth to knock out the SPARTIN gene in bone marrow mesenchymal stem cells (MSCs), thereby obtaining SPARTIN fl/fl; Prx1Cre mice.PCR analysis of genomic DNA from the ear or toe was used to confirm genotypes.

### 5.7 Femoral Bone Defects in Mice

The bone defect model was established in the femurs of mice using an electric bone drill. Prior to the experimental procedure, the mice were anesthetized via intraperitoneal injection and disinfected. Subsequently, the skin was incised, subcutaneous tissues were dissected to fully expose the femurs. For the creation of femoral defects, a 1.0‐mm drill bit was used. Two weeks after the operation, the femurs were harvested, and all samples were used for Micro‐CT detection and analysis.

### 5.8 Double Calcein‐Alizarin Red Labeling

Peritoneal injection with calcein (sigma, Cat.C0875) and Alizarin red (Merck, Cat.A5533) (30 mg·kg−1 body weight) was respectively performed at 10 and 3 days before mouse euthanasia. The tibias were harvested for undecalcified histology analysis. Femurs were collected and sectioned. Calcein‐Alizarin red labels were captured using a fluorescence microscope, and the MAR was calculated according to a previously reported standard by ImageJ.

### 5.9 Bone‐Targeting rAAV9‐eLIR Overexpression

Bone‐targeting rAAV9‐eLIR was designed and constructed as previously described.24 The DNA sequence encoding the bone‐specific peptide motif DSS (Asp‐Ser‐Ser)_6_ was inserted into the AAV9 capsid protein VP2 to build the rAAV9‐eLIR bone‐targeting overexpression vectors.

### 5.10 Immunofluorescence

Bone marrow mesenchymal stem cells (MSCs) at different differentiation stages were seeded onto confocal culture dishes at a density of 0.2×10^5^ cells/mL for culture. After cell adhesion, the cells were fixed with 4% PFA (neutral buffer, methanol‐free) for 20 min. For LC3B staining, the cells were treated with chloroquine (100 µM for 3 h; MCE, Cat. HY‐17589A) prior to fixation and staining.

After fixation, the cells were washed three times with 1× phosphate‐buffered saline (PBS), permeabilized with 0.5% Triton X‐100 for 20 min, and then blocked with PBS containing 10% fetal bovine serum (FBS) for 1 h. Following overnight incubation with the primary antibody against LC3B (Abmart, Cat. T55992S) or LAMP(Abcam, Cat.EPR21026) at 4°C, the samples were incubated with Goat Anti‐Rabbit IgG H&L (Alexa Fluor 555) (Abcam, Cat. ab150078) at room temperature for 1 h.

Subsequently, the cells were stained with a 10 µmol·L^−^^1^ BODIPY 493/503 solution (MCE, Cat.HY‐W090090 ) at room temperature for 1 h to label lipid droplets, followed by three washes with 1× PBS. Finally, the samples were mounted using DAPI antifade mounting medium (Beyotime, Cat. P0131), and images were acquired using a Zeiss LSM 900 confocal microscope. Statistics were processed using ImageJ.

### 5.11 Protein Extraction and Western Blot

Cell lysates were prepared using ice‐cold RIPA lysis buffer (Sigma–Aldrich, Cat. R0278). After lysis, whole‐cell proteins were extracted by centrifugation at 12,000 r·min^−^^1^ at 4°C for 30 min.

Proteins were separated by 10% SDS–PAGE gel electrophoresis and then transferred onto PVDF membranes (Merck Millipore, Cat. IPVH00010). The membranes were blocked with Tris‐buffered saline containing 5% non‐fat milk and Tween 20 (TBST), followed by overnight incubation with primary antibodies at 4°C. Subsequently, the PVDF membranes were incubated with horseradish peroxidase (HRP)‐conjugated anti‐mouse antibody (Cell Signaling Technology, Cat. 7076) or HRP‐conjugated anti‐rabbit antibody (Cell Signaling Technology, Cat. 7074) at room temperature for 1 h. Finally, the protein levels on the PVDF membranes were detected using a chemiluminescent reagent (Millipore, Cat. WBKLS0500).

The primary antibodies used in the experiment included: anti‐GAPDH (Cell Signaling Technology, Cat. 2118), anti‐SPARTIN (Proteintech, Cat. 13791‐1‐AP), anti‐CPT1A (Abcam, Cat. ab234111), and anti‐CPT2 (Cell Signaling Technology, Cat. 52552).

### 5.12 Micro‐CT Scanning

Micro‐CT scanning was performed using the Inveon MM system (Siemens) to evaluate bone tissue structure. The scanning parameters were set as follows: voxel size of 8.82 µm, voltage of 80 kV, current of 500 µA, exposure time of 1500 ms, and images were acquired layer by layer within a 360° rotation range.

The following parameters were calculated using the Inveon Research Workplace software (Siemens): bone volume fraction (BV/TV), trabecular thickness (Tb.Th), trabecular number (Tb.N), cortical thickness (Ct.Th), trabecular separation (Tb.Sp), and bone mineral density(BMD).

### 5.13 Intracellular ATP Measurement

The intracellular ATP level was measured using the ATP Assay Kit (Beyotime Biotechnology, S0027) by the bioluminescent method. The brief procedures were as follows: 2×10^4^ cells were seeded in 6‐well plates and cultured for 24 h. After washing with PBS, 200 µl of lysis buffer was added. Following lysis on ice for 30 min, the supernatant was collected by centrifugation at 12,000 g at 4°C for 5 min. 100 µl of ATP detection working solution was added to the assay tube and incubated at room temperature for 3–5 min to eliminate background ATP. Subsequently, 20 µl of sample or standard was added, and after rapid mixing, the relative light unit (RLU) value was measured using a chemiluminometer. All operations were performed strictly according to the manufacturer's instructions.

### 5.14 Intracellular Triglyceride Measurement

The triglyceride content was measured with the Triglyceride Determination Kit (Beyotime, Cat. S0219M). Relative triglyceride levels were presented as fold changes to the correspondent control, normalized against the protein content.

The neutrophil membrane was bought from the *Ruixibio* company (Wuhan, China) using the following procedure.

### 5.15 Isolation and In Vitro Aging of Peripheral Blood Neutrophils

Neutrophils were isolated from the whole blood of C57BL/6 mice using the Percoll density gradient centrifugation method. Blood was collected in heparinized tubes and centrifuged (400 × g, 10 min, 4°C). The resulting cell pellet was resuspended in PBS containing EDTA, carefully layered onto a three‐tiered Percoll gradient (52%, 69%, and 78%), and centrifuged at 1500 × g for 30 min at room temperature. Neutrophils were harvested from the enriched layer at the 69%/78% interface and the upper 78% gradient layer. The collected cells were subjected to red blood cell lysis at 4°C. To induce aging, the isolated neutrophils were cultured in vitro for 6 h.

### 5.16 Isolation of Neutrophil Membranes

Aged neutrophils were resuspended in ice‐cold Isolation Buffer‐1, with the following composition: 225 mM mannitol, 75 mM sucrose, 0.5% (w/v) bovine serum albumin, 0.5 mM EDTA, 30 mM Tris‐HCl, supplemented with a protease inhibitor cocktail. The cell suspension was homogenized on ice with a Dounce homogenizer for 50–100 strokes. The homogenate was centrifuged at 800 × g for 10 min at 4°C to pellet unbroken cells and nuclei. The resulting supernatant was further centrifuged at 10,000 × g for 10 min at 4°C to sediment mitochondria, and the pellet was discarded. The final supernatant was ultracentrifuged at 100,000 × g for 1 h at 4°C. The resulting neutrophil membrane pellet was washed with 10 mM Tris‐HCl and 0.5 mM EDTA buffer containing protease inhibitor cocktail, then freeze‐dried, weighed, and stored at −80°C for subsequent analysis.

### 5.17 Preparation of Digoxin‐Loaded PEGylated Liposomes

Digoxin‐loaded PEGylated liposomes (DGX‐LP) were prepared by thin‐film hydration. Briefly, L‐α‐phosphatidylcholine (lecithin), DSPE‐PEG2000, and cholesterol were mixed at a 5:1:5 (w/w/w) mass ratio in 3 mL chloroform. Digoxin was dissolved in 1 mL of methanol, combined with the lipid solution, and rotary‐evaporated to a thin film. The film was hydrated with ultrapure water under bath sonication, followed by polycarbonate membrane extrusion (200 nm pore size, Avanti Mini‐extruder) to obtain nanosized liposomes.

### 5.18 Neutrophil‐Membrane Coating

Purified neutrophil membranes were mixed with the liposomal cores at a 1:1 membrane‐to‐core (w/w) ratio, ice‐bath sonicated for 30 s, and incubated at room temperature for 2 h to allow membrane cloaking and vesicle fusion.

### 5.19 Purification and Lyophilization

Unencapsulated digoxin was removed by nano‐dialysis (polycarbonate membrane, 30 nm pore size; ND‐1 device). The purified formulations were lyophilized with a cryoprotectant and stored until use.

### 5.20 Physicochemical Characterization

Hydrodynamic size, polydispersity index (PDI), and ζ‐potential were measured by dynamic light scattering (DLS) and electrophoretic light scattering on a Brookhaven NanoBrook 90plus PALS. Morphology was examined by transmission electron microscopy (TEM; FEI TF20). Drug content was quantified by HPLC (Elite T3200) against a digoxin standard curve to calculate encapsulation efficiency (EE%) and drug loading (DL%). Serum stability and in vitro release were profiled under standard conditions as described (dialysis‐based sampling; details in Supplementary Methods), and all measurements were performed in ≥3 independent runs unless stated otherwise.

### 5.21 Statistics

GraphPad Prism (v10.4) was used for statistical analyses. For comparisons between two groups, unpaired Student's *t* tests were used Data are presented as the means ± SD ‘n’ in the article means biological replicate. We indicated significance as *ns*:no significance, ^*^ *p* < 0.05, ^**^ *p* < 0.01, ^***^ *p*< 0.001.

### 5.22 Ethics Approval and Consent to Participate

All participants provided informed consent, and the study was approved by the Ethics Committee of The Eighth Affiliated Hospital of Sun Yat‐Sen University with reference number: 2024‐236‐02. All animal experiments were conducted at the Laboratory Animal Center of Sun Yat‐sen University. All animal procedures were conducted in accordance with protocols approved by the Institutional Animal Care and Use Committee of Sun Yat‐Sen University with reference number: 2024002896.

## Conflicts of Interest

The authors declare no conflicts of interest.

## Supporting information




**Supporting File**: advs75348‐sup‐0001‐SuppMat.docx.

## Data Availability

The data that support the findings of this study are available in the supplementary material of this article.
