# Supplementary material for: Targeting Endogenous Lipophagy: A Novel Strategy to Enhance MSC Osteogenesis and Mineralization for Senile Osteoporosis Therapy
Source: Adv Sci (Weinh). 2026 Apr 27;13(39):e75348. doi: 10.1002/advs.75348 (PMC13335637; doi:10.1002/advs.75348)
Supplement: Supplementary file 1 — Supporting File: advs75348‐sup‐0001‐SuppMat.docx. [file ADVS-13-e75348-s001.docx]

Supporting Information

**Targeting Endogenous Lipophagy: A Novel Strategy to Enhance MSC Osteogenesis and Mineralization for Senile Osteoporosis Therapy**

*Chaoqiang Chen, Zhidong Liu, Yanhang Sun, Xiaojun Xu, Zhexiao Lan, Junhao Zhao, Peitao Xu*, Guiwen Ye* and Jinteng Li**

**Supplementary Figures**


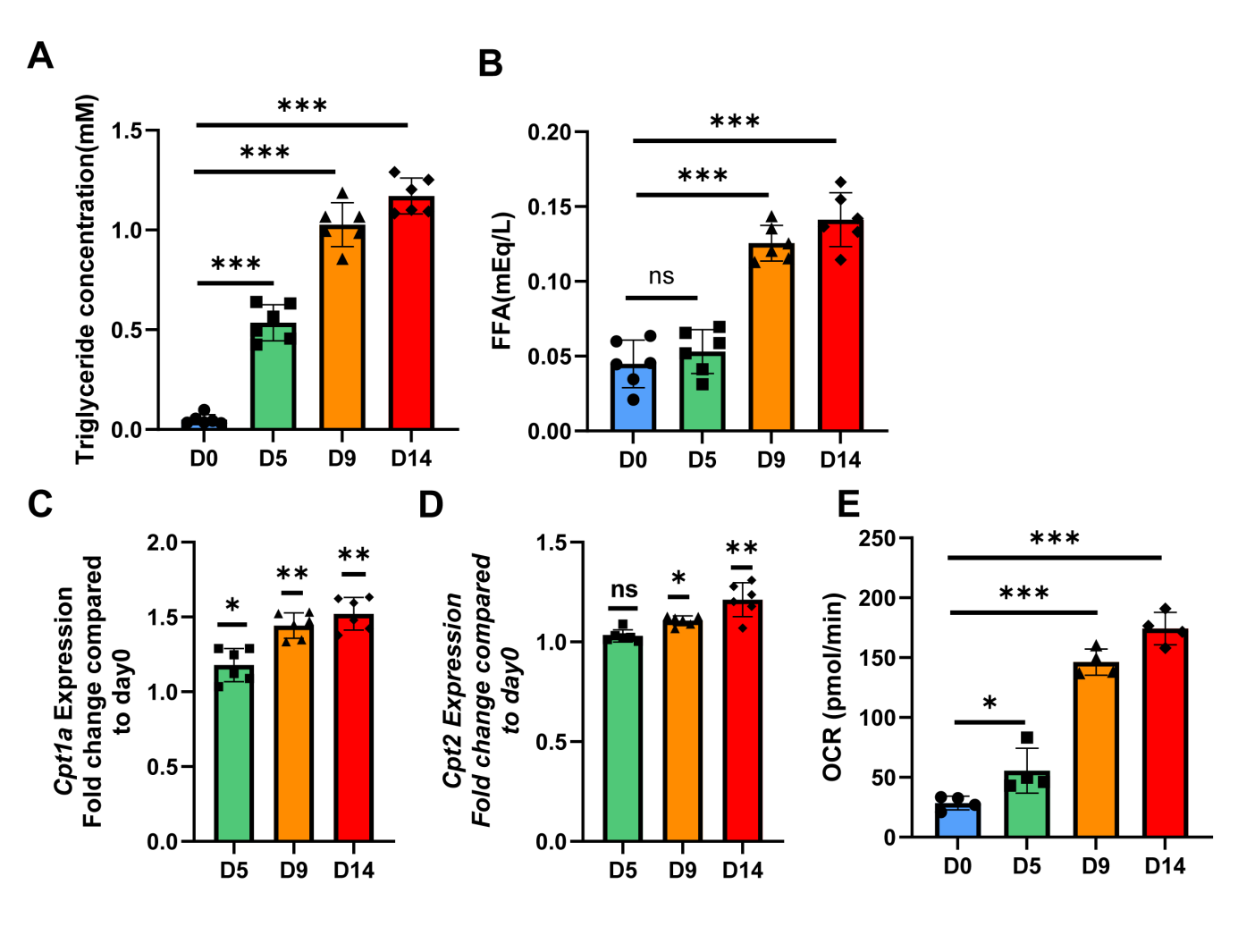


**Figure S1**

1. Intracellular triglyceride content in MSCs at different stages of osteogenic differentiation.(*n =* 6 )

B) Intracellular free fatty acid content in MSCs at different stages of osteogenic differentiation.(*n =* 6 )

C-D）Quantitative PCR analysis showing the relative mRNA expression levels of CPT1A and CPT2 in MSCs at different stages of osteogenic differentiation, normalized to day 0 .(*n =* 6 )

E) Quantitative analysis of the OCRs for MSCs at different stages of osteogenic differentiation.(*n =* 6 )

The Student’s t-test was used to analyze the differences between two groups and the data are expressed as mean ± SD,*ns*: no significance; **p* < 0.05*,**p* < 0.01*,***p <* 0.001.


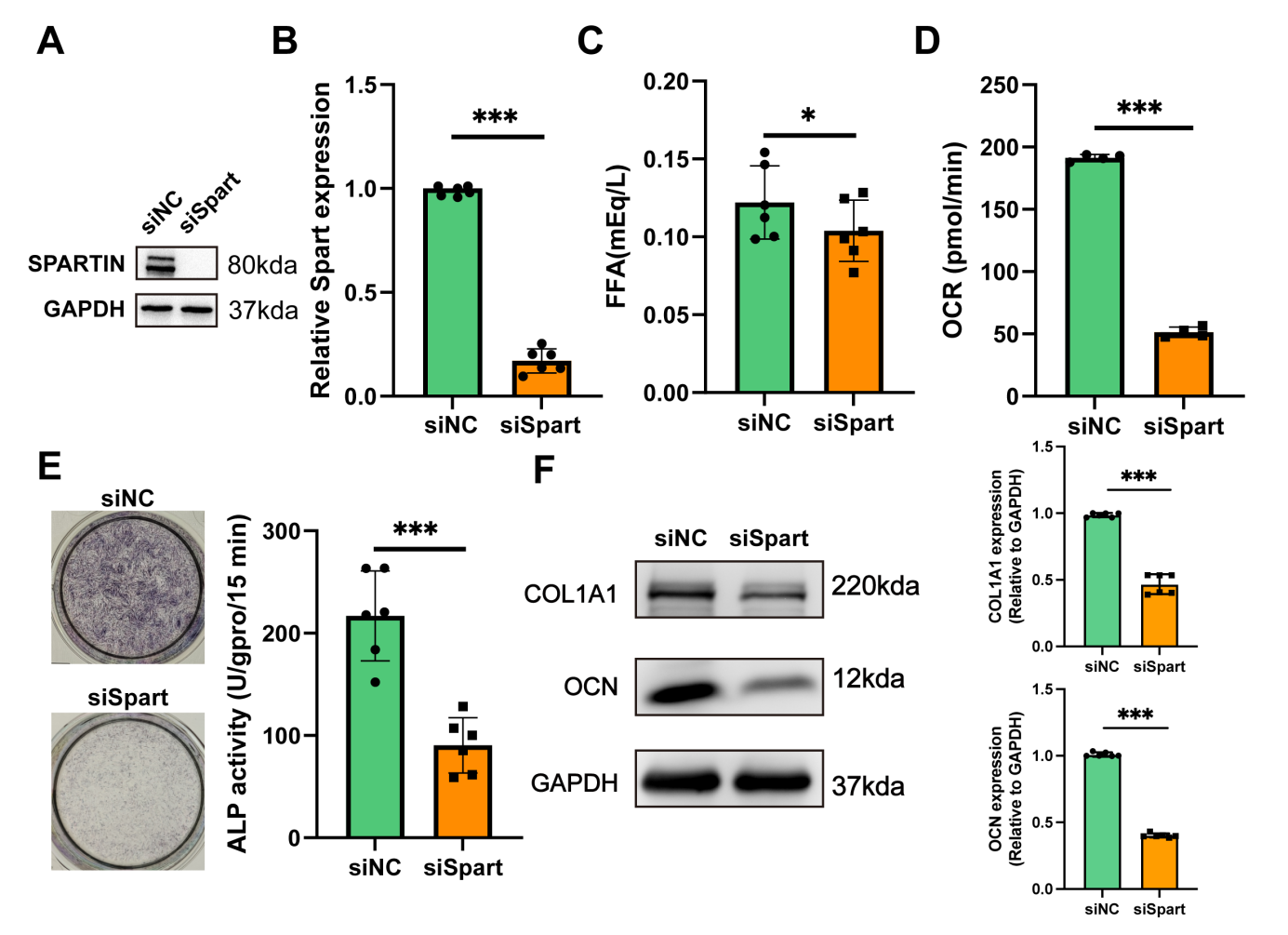


**Figure S2**

A-B) Representative Western blot(A) and qPCR(B) analyses demonstrating the efficient knockdown of Spart expression in MSCs following siSpart treatment.(*n =* 6 )

C) Decreased intracellular triglyceride content in MSCs after treated with siSpart. (*n =* 6 )

D) Quantitative analysis of the OCRs for MSCs after treated with siSpart. (*n =* 4 )

E) Representative ALP staining image and quantification of ALP activity in MSCs after treated with siSpart. (*n =* 6 )

F) Western blot analysis of OCN and COL1A1 protein expression in MSCs after treated with siSpart. (*n =* 6 )

The Student’s t-test was used to analyze the differences between two groups and the data are expressed as mean ± SD, *ns*: no significance; **p* < 0.05*,**p* < 0.01*,***p <* 0.001.


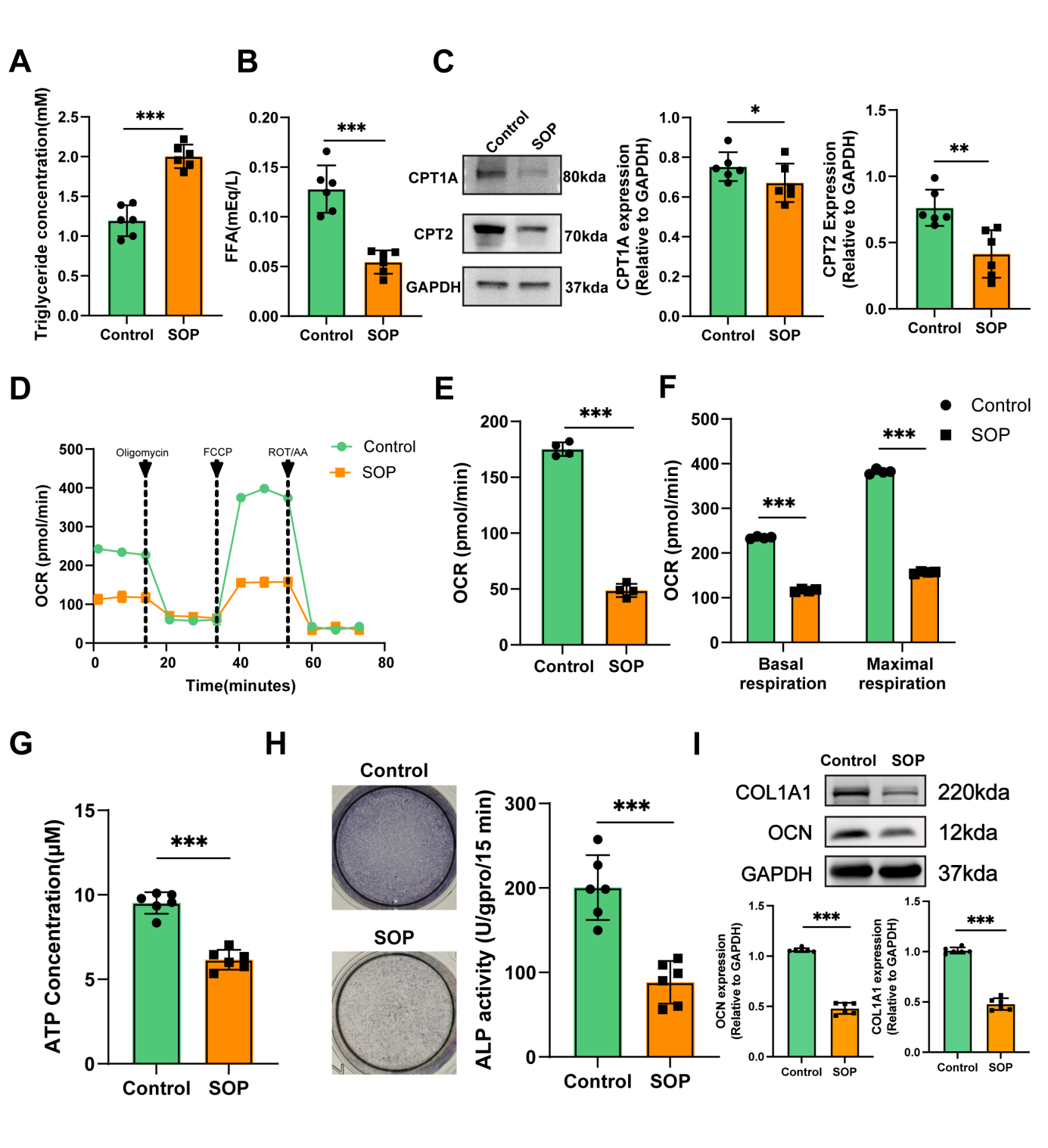


**Figure S3**

A) Quantification of intracellular triglyceride content showing elevated triglyceride accumulation in SOP-MSCs compared with controls (*n =* 6 ).

B) Quantification of intracellular free fatty acid levels showing reduced lipid hydrolysis in SOP-MSCs (*n =* 6 ).

C) Representative Western blot analysis and quantification of β-oxidation–related enzymes CPT1A and CPT2, showing decreased expression in SOP-MSCs (*n =* 6 ).

D) Representative Seahorse extracellular flux assay showing oxygen consumption rate curves of control and SOP-MSCs. (*n =* 4 ).

E-F) Quantification of overall OCR, basal respiration, and maximal respiration demonstrating impaired mitochondrial oxidative phosphorylation capacity in SOP-MSCs (*n =* 4 ).

G) Quantification of intracellular ATP concentration showing significantly reduced ATP levels in SOP-MSCs (*n =* 4 ).

H) Representative ALP staining image and quantification of ALP activity in SOP-MSCs. (*n =* 6 )

I) Western blot analysis of OCN and COL1A1 protein expression in SOP-MSCs. (*n =* 6 )

The Student’s t-test was used to analyze the differences between two groups and the data are expressed as mean ± SD, *ns*: no significance; **p* < 0.05*,**p* < 0.01*,***p <* 0.001.


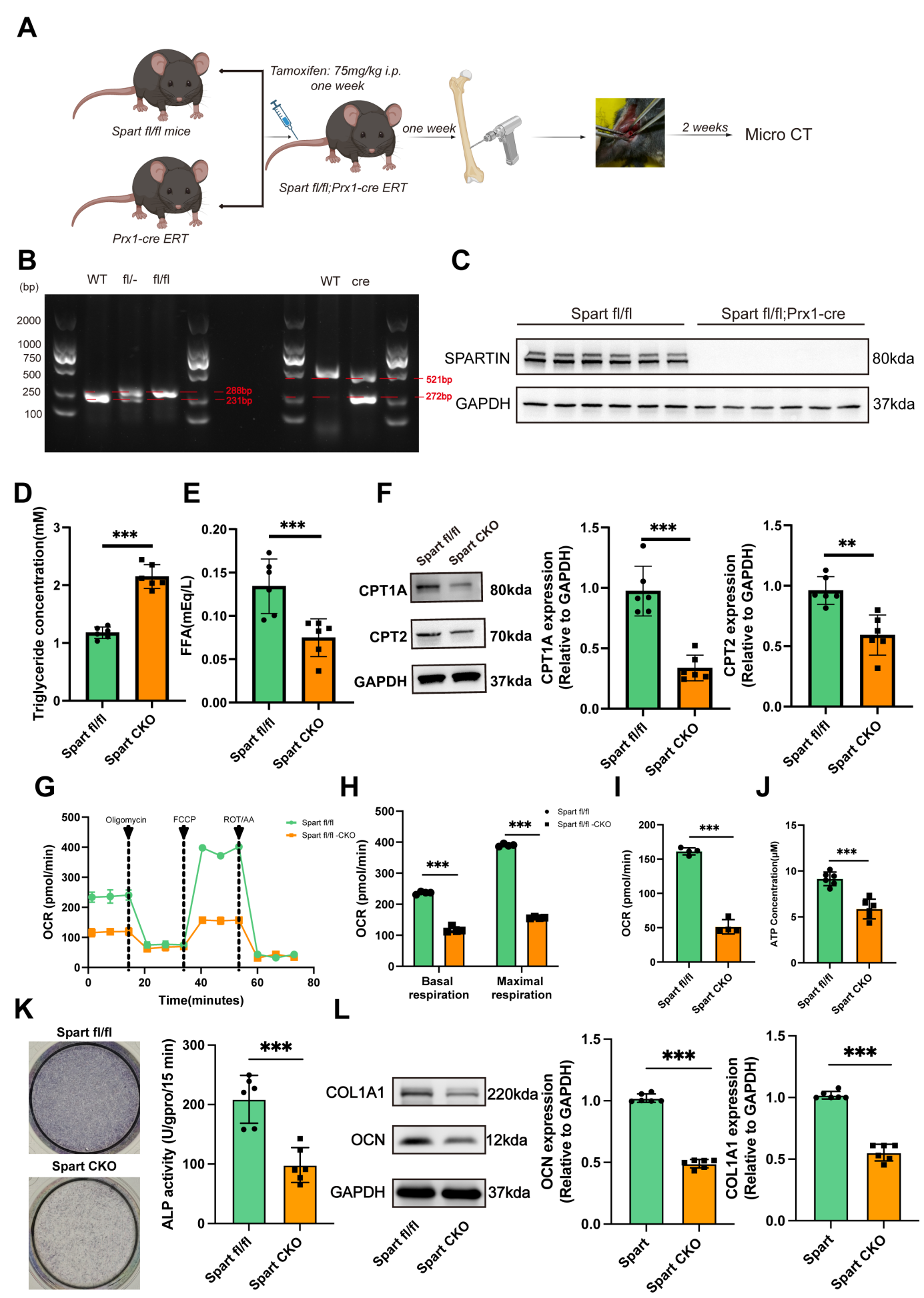


**Figure S4**

A) Schematic illustration of the generation of Spart conditional knockout (CKO) mice using the Prx1-CreERT2 system. Spart fl/fl mice were crossed with Prx1-CreERT2 mice to achieve MSC-specific deletion of SPARTIN upon tamoxifen induction. Mice were analyzed by micro-CT one week after tamoxifen administration.

B) DNA electrophoresis was performed to genotype genetically modified mice.

C) Representative Western blot confirming efficient depletion of SPARTIN protein in MSCs from Spart fl/fl;Prx1-CreERT2 mice (Spart CKO).

D-E) Quantification of intracellular triglyceride and free fatty acid levels showing elevated TG accumulation and reduced FFA content in MSCs from Spart CKO mice (*n =* 6 ).

F) Representative immunoblots and quantification showing decreased expression of fatty acid β-oxidation–related enzymes CPT1A and CPT2 in MSCs from Spart CKO mice (*n =* 6 )

G) Representative Seahorse extracellular flux assay showing oxygen consumption rate curves of control and Spart CKO MSCs. (*n =* 4 ).

H-I) Quantification of overall OCRs, basal and maximal respiration demonstrating reduced mitochondrial oxidative phosphorylation in Spart CKO MSCs (*n =* 4 ).

J) Quantification of intracellular ATP concentration showing significantly reduced ATP production in Spart CKO MSCs (*n =* 6 ).

K) Representative ALP staining image and quantification of ALP activity in Spart CKO MSCs. (*n =* 6 )

L) Western blot analysis of OCN and COL1A1 protein expression in Spart CKO MSCs. (*n =* 6 )

The Student’s t-test was used to analyze the differences between two groups and the data are expressed as mean ± SD, *ns*: no significance; **p* < 0.05*,**p* < 0.01*,***p <* 0.001.


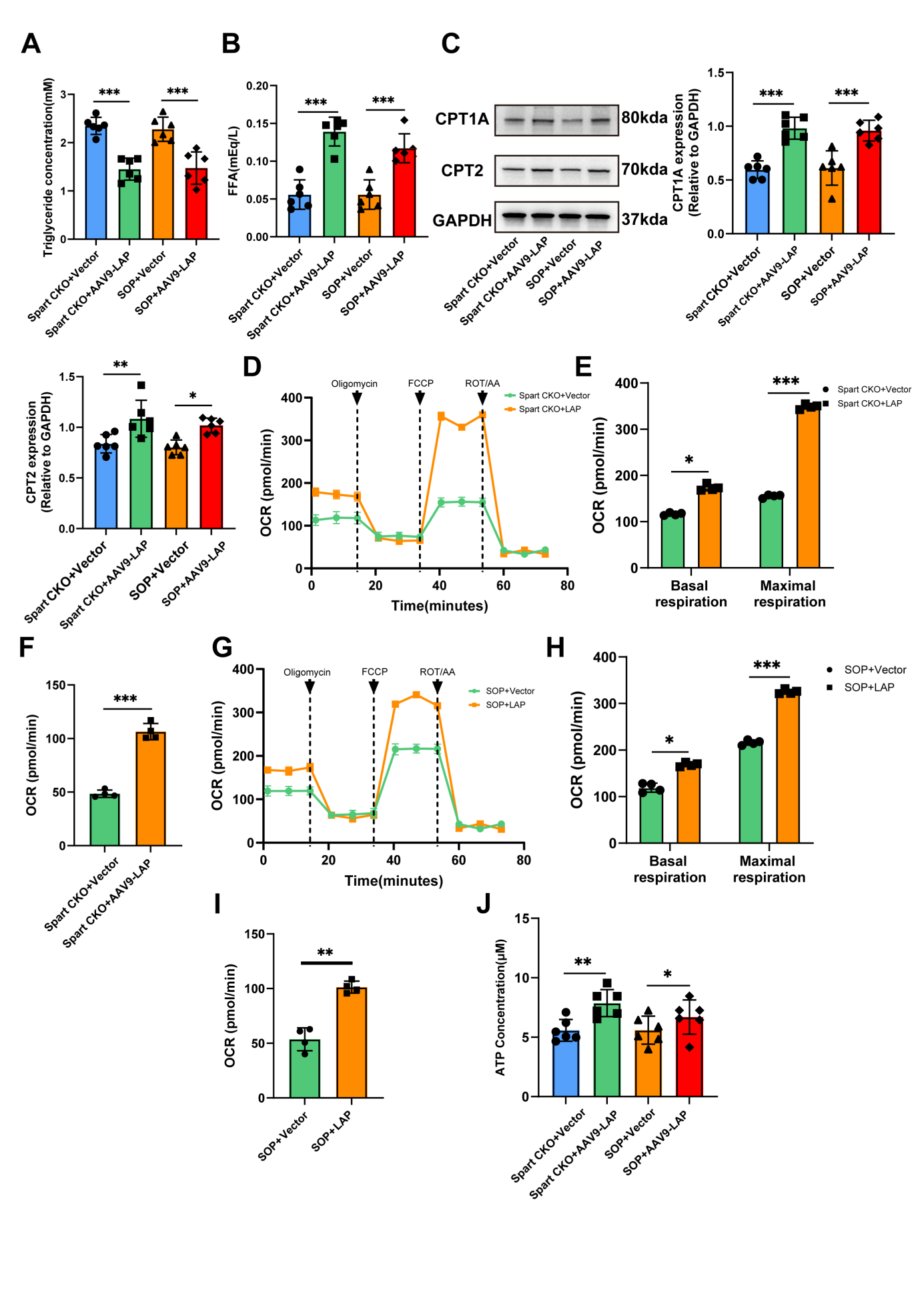


**Figure S5**

A-B) Quantification of intracellular triglyceride and free fatty acid levels showing that btAAV9-LAP treatment reduced TG accumulation and increased FFA content in Spart CKO MSCs (*n =* 6 ).

C) Representative Western blot and quantitative analysis showing partial restoration of fatty-acid β-oxidation–related enzymes CPT1A and CPT2 expression in MSCs from btAAV9-LAP–treated mice (*n =* 6 ).

D) Representative Seahorse extracellular-flux assay showing oxygen-consumption-rate curves of vector- and btAAV9-LAP–treated Spart CKO MSCs (*n =* 4 )

E-F) Quantification of overall OCR, basal and maximal respiration showing improved mitochondrial oxidative-phosphorylation capacity in btAAV9-LAP–treated MSCs (*n =* 4 ).

G) Quantification of intracellular ATP concentration showing significantly increased ATP production following btAAV9-LAP treatment (*n =* 6 )

The Student’s t-test was used to analyze the differences between two groups, and the data are expressed as mean ± SD. *ns*: no significance; **p* < 0.05*,**p* < 0.01*,***p <* 0.001.


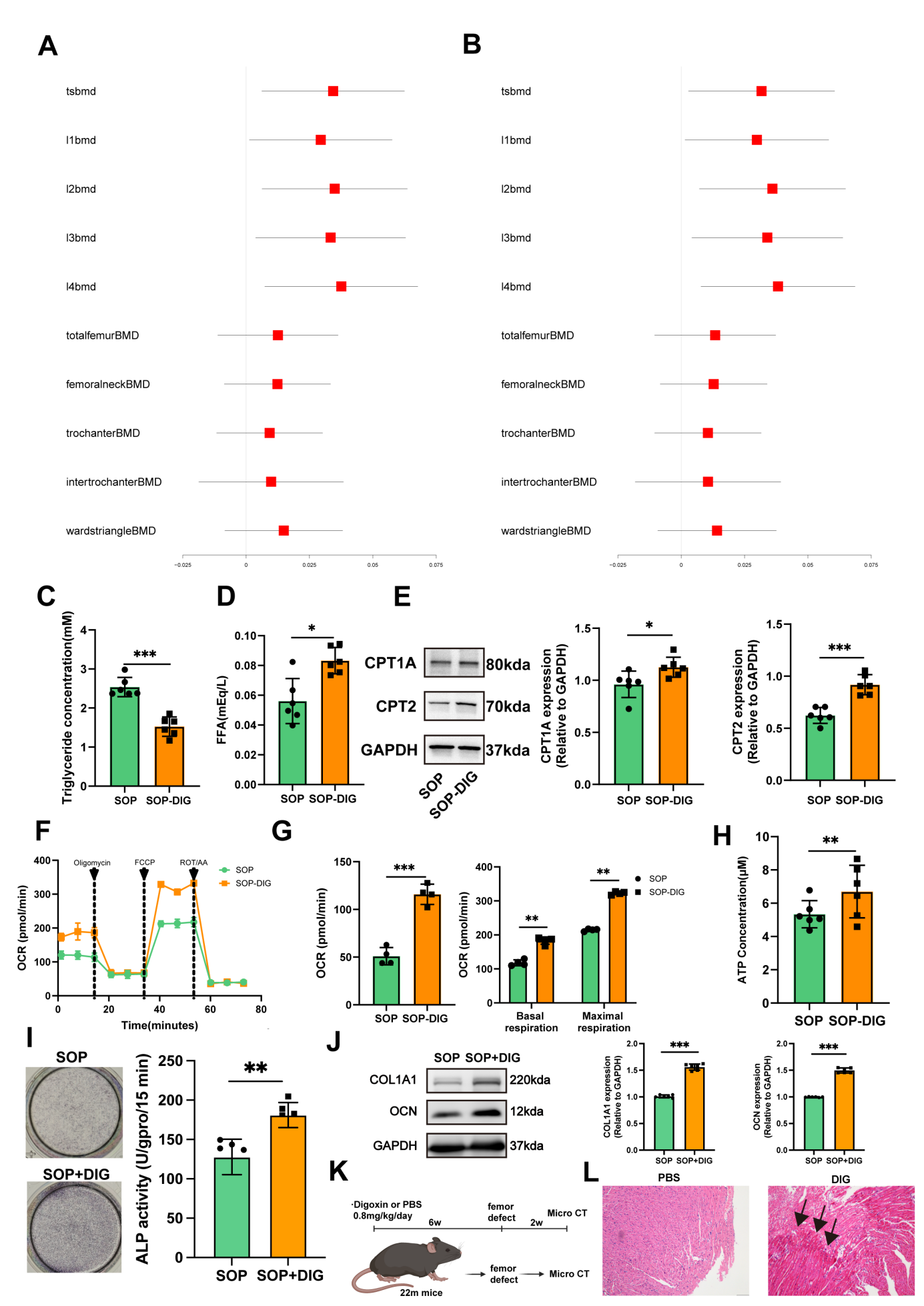


**Figure S6**

A-B) Forest plots showing regression coefficients from multivariate linear models assessing the association between digoxin use and BMD using NHANES 2005–2018 data (201 digoxin users ≥ 65 years vs 367 matched controls). Model 1 adjusted for age, race, and gender; Model 2 further adjusted for smoking, BMI, CKD, and fracture history. Digoxin use was positively associated with total and lumbar (L1–L4) BMD in both models.

C) Quantification of intracellular triglyceride levels showing reduced TG accumulation in MSCs isolated from digoxin-treated (SOP-DIG) mice compared with untreated SOP controls (*n =* 6 ).

D) Quantification of intracellular free fatty acid levels showing elevated FFA in MSCs from DIG-treated SOP mice compared with untreated controls (*n =* 6 ).

E) Representative Western blots and quantitative analysis showing elevated expression of fatty acid β-oxidation–related enzymes CPT1A and CPT2 in SOP-DIG MSCs compared with untreated SOP MSCs (*n =* 6 )

F) Representative Seahorse extracellular-flux analysis showing oxygen-consumption-rate curves of MSCs from SOP and DIG-treated SOP mice (*n =* 4 ).

G) Quantification of overall OCR, basal and maximal respiration demonstrating improved mitochondrial oxidative-phosphorylation capacity following DIG treatment (*n =* 4 ).

H) Quantification of intracellular ATP concentration showing significantly increased ATP production in MSCs from SOP-DIG mice (*n =* 4 ).

I) Representative ALP staining image and quantification of ALP activity in MSCs from SOP-DIG mice. (*n =* 6 )

J) Western blot analysis of OCN and COL1A1 protein expression in MSCs from SOP-DIG mice. (*n =* 6 )

K) Schematic illustration of the in-vivo experimental design for digoxin administration in 22-month SOP mice (0.8 mg/kg/day for 6 weeks), followed by femoral-defect surgery and micro-CT analysis after 2 weeks.

L) Representative histological images showing that DIG treatment induces myocardial injury in mice(black arrow)

The Student’s t-test was used to analyze the differences between two groups, and data are expressed as mean ± SD. *ns*: no significance; **p* < 0.05*,**p* < 0.01*,***p <* 0.001.

**
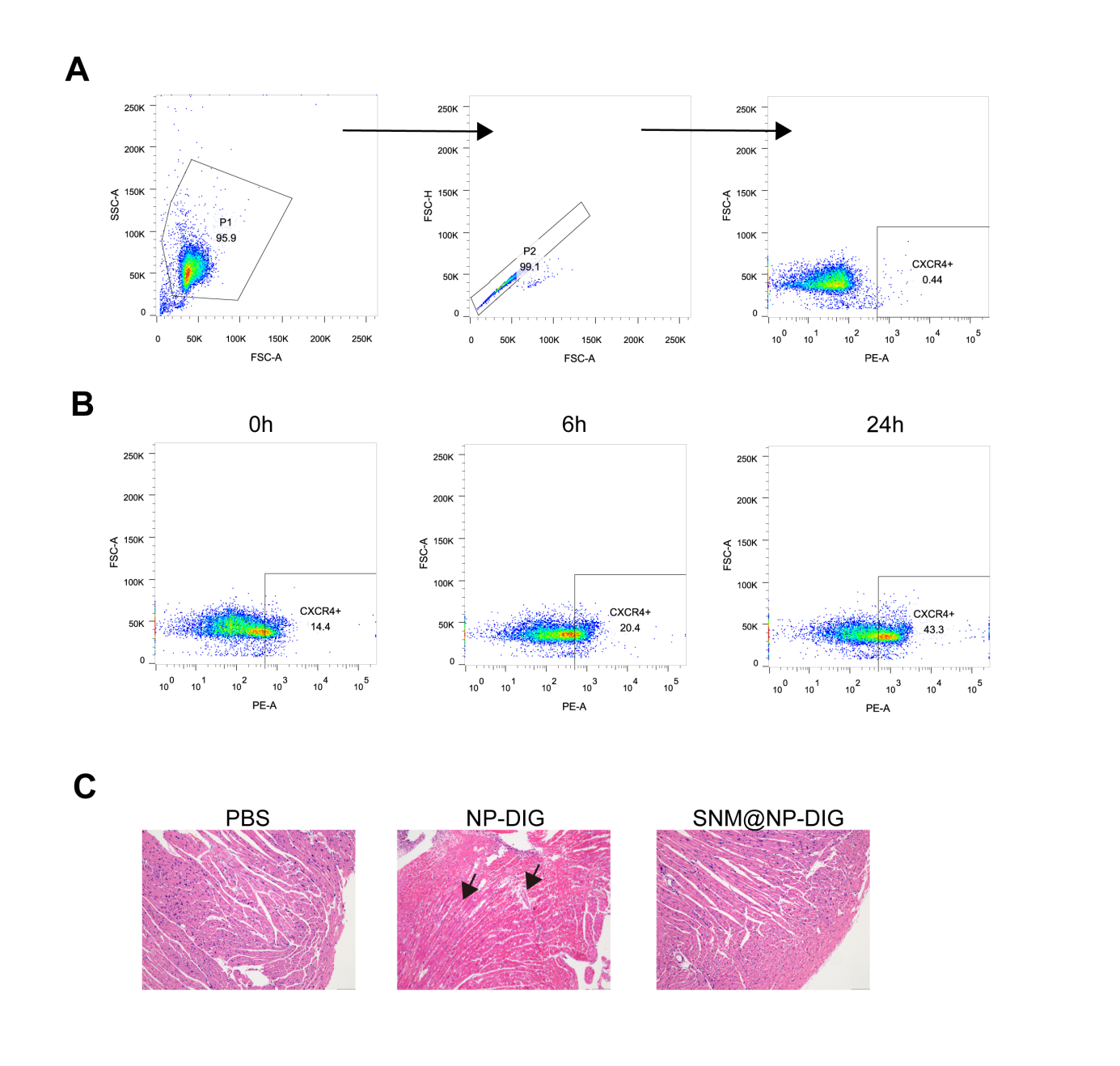
**

**Figure S7**

A-B) Flow cytometric analysis showing dynamic changes in CXCR4 expression on neutrophils at different time points in vitro.

C) Representative images showing that SNM@NP-DIG treatment attenuates myocardial injury.

**Supplementary Tables**

**Table S1. The baseline of NHANES database in article**

| **Variable** |  | **ALL (N=568)** | **Control (N=367)** | **Digoxin (N=201)** | **p.overall** |
| --- | --- | --- | --- | --- | --- |
| **Age** |  | 73.1 (8.12) | 72.9 (8.22) | 73.5 (7.94) | 0.387 |
| **Race** |  |  |  |  | 0.389 |
|  | Mexican American | 34 (5.99%) | 22 (5.99%) | 12 (5.97%) |  |
|  | NonHispanic White | 381 (67.1%) | 238 (64.9%) | 143 (71.1%) |  |
|  | NonHispanic Black | 118 (20.8%) | 84 (22.9%) | 34 (16.9%) |  |
|  | Other | 35 (6.16%) | 23 (6.27%) | 12 (5.97%) |  |
| **Gender** |  |  |  |  | 0.377 |
|  | Male | 335 (59.0%) | 211 (57.5%) | 124 (61.7%) |  |
|  | Female | 233 (41.0%) | 156 (42.5%) | 77 (38.3%) |  |
| **Smoke status** |  |  |  |  | 0.015 |
|  | Yes | 290 (51.1%) | 173 (47.1%) | 117 (58.2%) |  |
|  | No | 278 (48.9%) | 194 (52.9%) | 84 (41.8%) |  |
| **BMI** |  | 28.1 (5.90) | 28.2 (6.03) | 28.1 (5.68) | 0.818 |
| **CKD** |  |  |  |  | <0.001 |
|  | Yes | 24 (4.23%) | 5 (1.36%) | 19 (9.45%) |  |
|  | No | 544 (95.8%) | 362 (98.6%) | 182 (90.5%) |  |
| **Hip fracture** |  |  |  |  | 0.435 |
|  | Yes | 17 (2.99%) | 13 (3.54%) | 4 (1.99%) |  |
|  | No | 551 (97.0%) | 354 (96.5%) | 197 (98.0%) |  |
| **Wrist fracture** |  |  |  |  | 0.858 |
|  | Yes | 59 (10.4%) | 37 (10.1%) | 22 (10.9%) |  |
|  | No | 509 (89.6%) | 330 (89.9%) | 179 (89.1%) |  |
| **Spine fracture** |  |  |  |  | 0.154 |
|  | Yes | 21 (3.70%) | 10 (2.72%) | 11 (5.47%) |  |
|  | No | 547 (96.3%) | 357 (97.3%) | 190 (94.5%) |  |

**Table S2.** Sequences of siRNA used in RNA Interference assay

| siRNA |  | Sequence |
| --- | --- | --- |
| Spart | Forward | 5'-GAGGGCCACUACACUGUUUTT-3' |
|  | Reverse | 5'-AAACAGUGUAGUGGCCCUCTT-3' |

**Table S3.** Sequences of primers used in RT-qPCR assay

| Gene |  | Sequence |
| --- | --- | --- |
| Spart | Forward | 5'-ATCCCTGGGAGATCAAGTCAC-3' |
|  | Reverse | 5'-CTGCCTTGGTCTATTGAGGAAC-3' |
| Gapdh | Forward | 5'-AGCCATCAGCTATGCACGTA-3' |
|  | Reverse | 5'-ACCGTGCTATAAAAGCCCTGC-3' |
| Spart flox/flox | Forward | 5'-CGTTGGAGACCAGAGGTCAG-3' |
|  | Reverse | 5'-GGTAGCAGTAAAGAGGGCAGG-3' |
| Prrx1-cre | Forward | 5'-AGCGATGGATTTCCGTCTCTGG-3' |
|  | Reverse | 5'-AGCTTGCATGATCTCCGGTATTGAA-3' |

**Table S4.** Information of antibodies used in western blot analysis and flow metry analysis.

| Antibodies | Company | Catalog number |
| --- | --- | --- |
| PC anti-mouse CD184 (CXCR4) Antibody | BioLegend | #146507 |
| Anti-GAPDH | Cell Signaling Technology | #2118 |
| Anti-SPARTIN | Proteintech | #13791-1-AP |
| Anti-CPT1A | Abcam | #ab234111 |
| Anti-CPT2 | Cell Signaling Technology | #52552 |
| Anti-LC3B | Abmart | #T55992S |
| Anti-LAMP1 | Abcam | #EPR21026 |
